# Reliability Analysis of AlGaN-Based Deep UV-LEDs

**DOI:** 10.3390/nano12213731

**Published:** 2022-10-24

**Authors:** Mudassar Maraj, Li Min, Wenhong Sun

**Affiliations:** 1 Research Center for Optoelectronic Materials and Devices, Guangxi Key Laboratory for the Relativistic Astrophysics, School of Physical Science and Technology, Guangxi University, Nanning 530004, China; 2 MOE Key Laboratory of New Processing Technology for Non-Ferrous Metals and the Guangxi Key Laboratory of Processing for Non-Ferrous Metals and Featured Materials, Guangxi University, Nanning 530004, China

**Keywords:** GaN, AlGaN, near UV-LEDs, degradation, reliability, SARS-CoV-2 disinfection technology

## Abstract

The current pandemic crisis caused by SARS-CoV-2 has also pushed researchers to work on LEDs, especially in the range of 220–240 nm, for the purpose of disinfecting the environment, but the efficiency of such deep UV-LEDs is highly demanding for mass adoption. Over the last two decades, several research groups have worked out that the optical power of GaN-based LEDs significantly decreases during operation, and with the passage of time, many mechanisms responsible for the degradation of such devices start playing their roles. Only a few attempts, to explore the reliability of these LEDs, have been presented so far which provide very little information on the output power degradation of these LEDs with the passage of time. Therefore, the aim of this review is to summarize the degradation factors of AlGaN-based near UV-LEDs emitting in the range of 200–350 nm by means of combined optical and electrical characterization so that work groups may have an idea of the issues raised to date and to achieve a wavelength range needed for disinfecting the environment from SARS-CoV-2. The performance of devices submitted to different stress conditions has been reviewed for the reliability of AlGaN-based UV-LEDs based on the work of different research groups so far, according to our knowledge. In particular, we review: (1) fabrication strategies to improve the efficiency of UV-LEDs; (2) the intensity of variation under constant current stress for different durations; (3) creation of the defects that cause the degradation of LED performance; (4) effect of degradation on C-V characteristics of such LEDs; (5) I-V behavior variation under stress; (6) different structural schemes to enhance the reliability of LEDs; (7) reliability of LEDs ranging from 220–240 nm; and (8) degradation measurement strategies. Finally, concluding remarks for future research to enhance the reliability of near UV-LEDs is presented. This draft presents a comprehensive review for industry and academic research on the physical properties of an AlGaN near UV-LEDs that are affected by aging to help LED manufacturers and end users to construct and utilize such LEDs effectively and provide the community a better life standard.

## 1. Introduction

While designing an electronic system, it is important to consider and specify the warranty type that has to be provided to the consumers. To ensure this practice, the life of the device should be tested without failure. It is mandatory for the LED industry to provide the guaranteed lifespan of LEDs in working condition for the end users of the products that utilize LEDs. This information helps the designer in optimizing the cost and efficiency of the end product. It also enables the manufacturer to provide the best combination of lighting performance, ownership cost, and purchase price. The integration of LEDs in conventional applications is hindered due to relatively meager and sporadic information of their reliability. Depending upon the application and manufacturing LEDs, typical lifespan of the LEDs varies between 3 months and 50,000–70,000 h [1]. Lumen maintenance is employed to gauge the lifetime of an LED. It shows the fall in the intensity of emitted light with time. LEDs with 50% and 70% degradation of output light are labeled L50 and L70, respectively [2]. The former is the best fit for the display industry approach, whereas the latter is suitable for use in the lighting industry. These light output terms for degradations of LED at room temperature are defined by the Alliance for Solid-State Illumination Systems and Technologies (ASSIST) [2].

**Figure 1:**
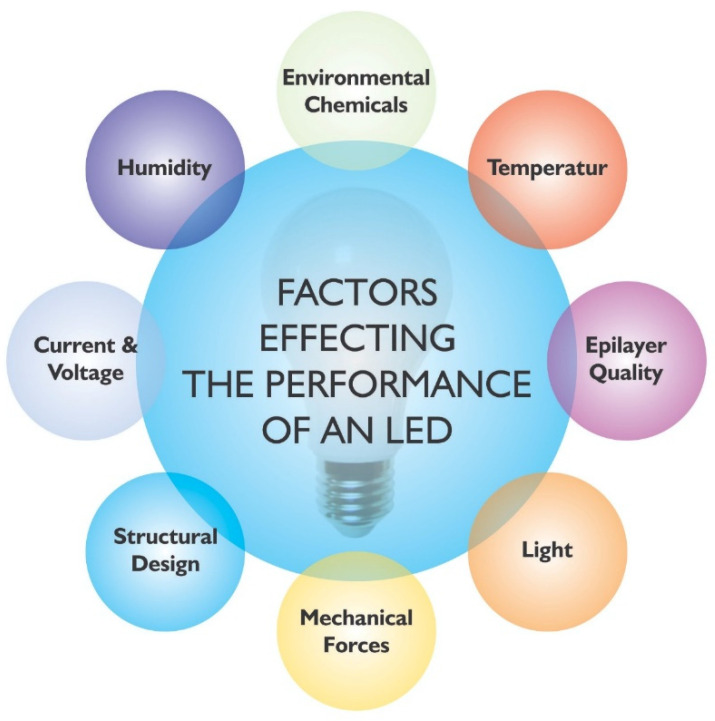
Factors effecting LED reliability.

The lifetime of LED light sources is also influenced by various factors, and the most effective factors are current and voltage, humidity, temperature, chemicals and mechanical forces, light radiation etc. as depicted in Figure 1.

However, these factors can cause a total failure of the device or affect its working in the long run, if not adjusted properly. That is how these factors influence the reliability as well as the lifetime in the worst-case scenario.

Due to the high efficiency (for white LEDs, >150 Lm/W [1]) and longer lifespan, gallium nitride (GaN)-based light-emitting diodes (LEDs) stand in for the almost ideal light sources of the next generation. By carefully controlling the compositional properties of the material, customized wavelength emission is made possible. Moreover, the cost of ownership is expectedly low for this particular scenario. Many particular fields, such as display backlighting, solid-state lighting, automotive, and portable systems require light sources with high performance rates and improved reliability. Numerous researchers worldwide have improved the performance of visible LEDs for their relevant integration in the above mentioned diverse fields [3].AlGaN alloy is determined to have a tunable direct bandgap (3.4–6.11 eV). This is found to be suitable material for the manufacturing of optical devices [4,5,6,7]. In the past, mercury-based lamps were employed predominantly for these applications. On 16 August 2017, the convention on mercury took place, and mercury lamps were contemplated to be substituted by LEDs based on AlGaN [8].

However, the emission spectrum utility of nitride materials of group III is minute. The intermixing of GaN and AlN materials gives rise to AlGaN-based LEDs with ultraviolet (UV) in the entire spectral range of 210–400 nm. UV-LED devices can be used in an extensive variety of applications, such as environmental sensing, UV curing, phototherapy, water purification, UV curing, and plant growth lighting, all regulated by emission wavelength [9].

**Figure 2:**
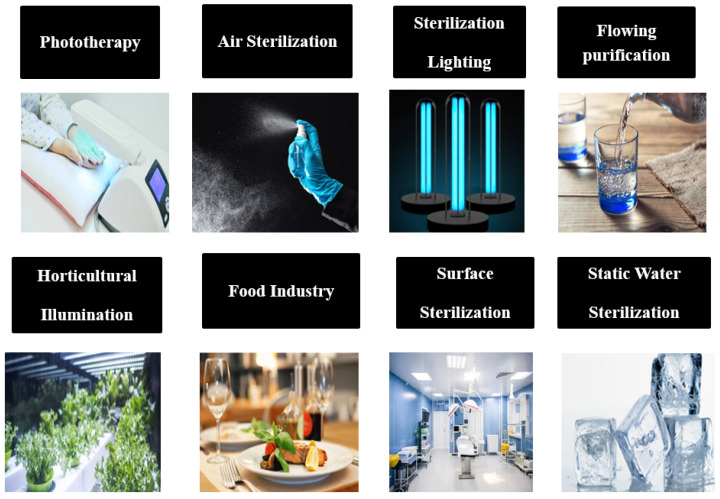
Application of near UV-LEDs.

In terms of performance, UV-LEDs are not as good as visible LEDs; however, this new technology holds prodigious potential for utilization in many fields. The underexplored ranges of the electromagnetic (EM) spectrum include the near-ultraviolet (DUV) range, which is attributed to the radiation having a shorter wavelength (<350 nm). Such devices possess distinct properties, such as low noise, space power, and spectral distribution, and high modulation frequency comparable with conventional mercury lamps to a great extent [10]. These newly discovered UV sources have found potential applications in diverse areas of biomedical sciences [11]. Figure 2 illustrates some of the possible applications of UV-LEDs.

The reliability of these devices has not yet been established. Only limited work has been published until now. Very little work has been done on the exploration of gradual degradation of output light, for that reason, this review focuses on analyzing the degradation mechanism of DUV light-emitting diodes by incorporating electrical and optical characterization within the range of 200–350 nm. The performance of devices that were subjected to different stress conditions have been reviewed for the reliability of AlGaN-based UV-LEDs worked out by different research groups so far according to our knowledge. In particular, we review: (1) AlGaN UV-LED fabrication for improved efficiency; (2) intensity variation under constant current stress for different durations; (3) creation of the defects that cause the degradation of LED performance; (4) effect of degradation on C-V characteristics of such LEDs; (5) I-V behavior variation under stress; (6) different approaches to enhance the reliability of LEDs; (7) reliability of LEDS with wavelengths ranging from 220–240 particularly important for SAR-CoV 2 disinfection. Finally, concluding remarks for future researchers to enhance the reliability of near UV-LEDs is presented.

## 2. Fabrication of DUV-LEDs

The light emission from AlGaN light-emitting diodes can be tuned to approximately cover all the ultraviolet spectral range of wavelength (210–400 nm) and this tuning makes UV-LEDs fascinatingly suitable to a wide number of industrial, environmental, biological or medical applications. Apart from their broad range of applications, the near UV-LEDs still have some flaws, such as low external quantum efficiency (EQE) due to Al-rich group-III nitride elements [9]. Therefore, to improve the efficiency of near UV-LEDs different modification schemes have been employed so far by different research groups, which are compared later in this section. The improved efficiency is based on the improved quality of fabrication to address the different issues that hinder their performance.

**Figure 3:**
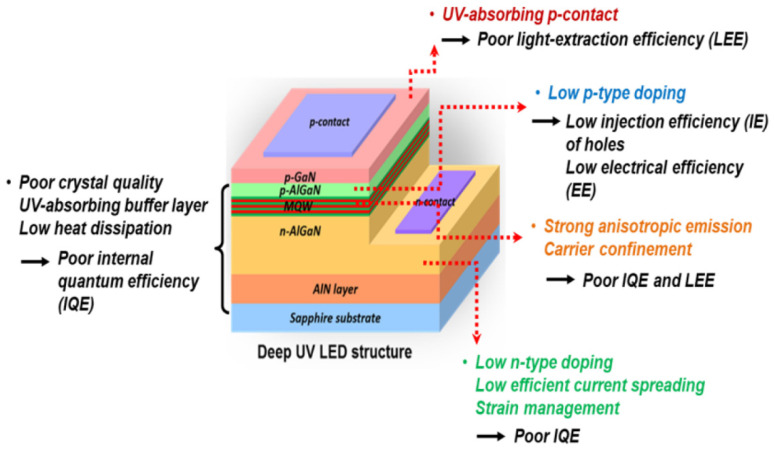
The schematic diagram of a typical UV-LED and related physical phenomenon associated with different layered structures. [12] Copyright 2021, MDPI Publishing.

A typical lateral structure LED chip schematic diagram is shown in Figure 3 [12]. The different techniques that are employed for such fabrication of UV-LED chips are thin-film deposition techniques, photolithography, thermal annealing, etching process, grinding and polishing. Among these techniques, the lateral structure UV-LED chip fabrication mainly involves photolithography, etching, sputtering, and metal evaporation. This lateral structure has a specialty in that the p-type and n-type electrodes of the gallium nitride based UV-LEDs chips are fabricated epitaxially on the same side of substrate, which is commonly a sapphire substrate and mostly chosen due to its highly stable physical and chemical properties [13].

According to Figure 3, gallium nitride-based LED chips are based on a sapphire substrate, an n-type doped layer (n-GaN), a stack of multiple quantum wells, a p-type doped layer (p-GaN), followed by an indium tin oxide (ITO) transparent conductive layer then the metal contacts. For the fabrication of the lateral structure LED chip, the ohmic metal contact is placed on the top of the p-GaN epitaxial layer, thereby forming a p-type electrode layer. The p-GaN and MQWs are first etched then the metal contacts are deposited on n-GaN [13].

The different performance factors caused by the different layers involved in the fabrication of UV-LEDs are also highlighted in Figure 3. The main parameters that ensure the better performance of UV-LEDs are the external quantum efficiency, the wall-plug efficiency (WPE) and the optical output power *P_out_*. The values of these parameters for near UV-LEDs are considerably less competent than visible LEDs. The reasons for the low efficiency come from low conductivity of semiconductor heterostructure interfaces, poor ultraviolet light reflectivity of n- and p-contacts and poor transparency of semiconductor layers to UV light [14], So UV-LEDs still offer a wide space for improvement and very elementary and necessary development consists of aggregate light extraction efficiency (LEE) of near UV-LEDs. Most of the UV-LEDs heterostructures are grown on sapphire substrates that provide a low-cost template for ultraviolet emitters, but with a larger lattice mismatch of about 14% between sapphire and aluminum nitride surface, resulting in the creation of several dislocations at the sapphire–AlN interface. One of the major challenges is the comparatively high operating voltages of gallium nitride based UV-LEDs, which causes the poor electrical conductivities of Si- and Mg-doped AlGaN layers [9].

**Figure 4:**
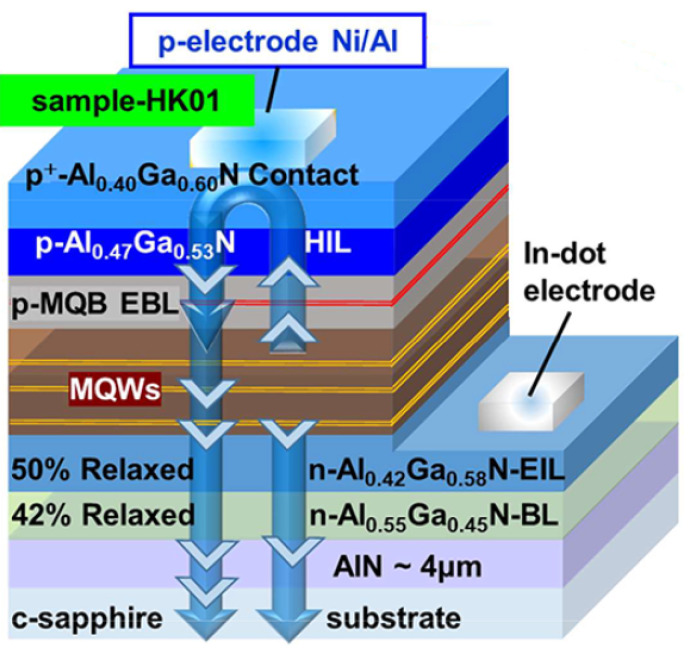
Schematic view of EIL-based 310 nm band UVB LEDs based on highly reflective Ni/Al p-electrode. [16] Copyright 2020, ACS Publishing.

Although, there has been a significant improvement in fabricating of UV-LEDs with improved quantum efficiencies, both internal and external and with low density of threading dislocations but the effort is still going on to meet the market standards. Muhammad Usman et al. numerically investigated AlGaN-based UVB LEDs for the suppression of efficiency droop and got enhanced hole injection in MQWs [15]. Their numerical simulation suggested that, compared to the conventional structure, the proposed structure had a high peak efficiency and very small efficiency droop. Muhammad Ajmal et al. resolved the issue of nonlinearity in the light output power *P_out_* and EQE (*η_ext_*) of 310 nm band UVB LEDs. They got *η_ext_* up to 4.7% and *P_out_* up to 29 mW. By increasing the Al contents from 48% to 55% in the undoped AlGaN, for the 294 nm band UVB LED, the Al-content difference also increased from 15% to 20%, the *P_out_* was greatly enhanced from 17 mW to 32 mW. The *η_ext_* was also improved from 5.6 to 6.5% using the Ni (1 nm)/Al (200 nm) p-electrode and insertion of moderately Mg-doped p-MQB EBL, as shown in Figure 4 [16].

**Figure 5:**
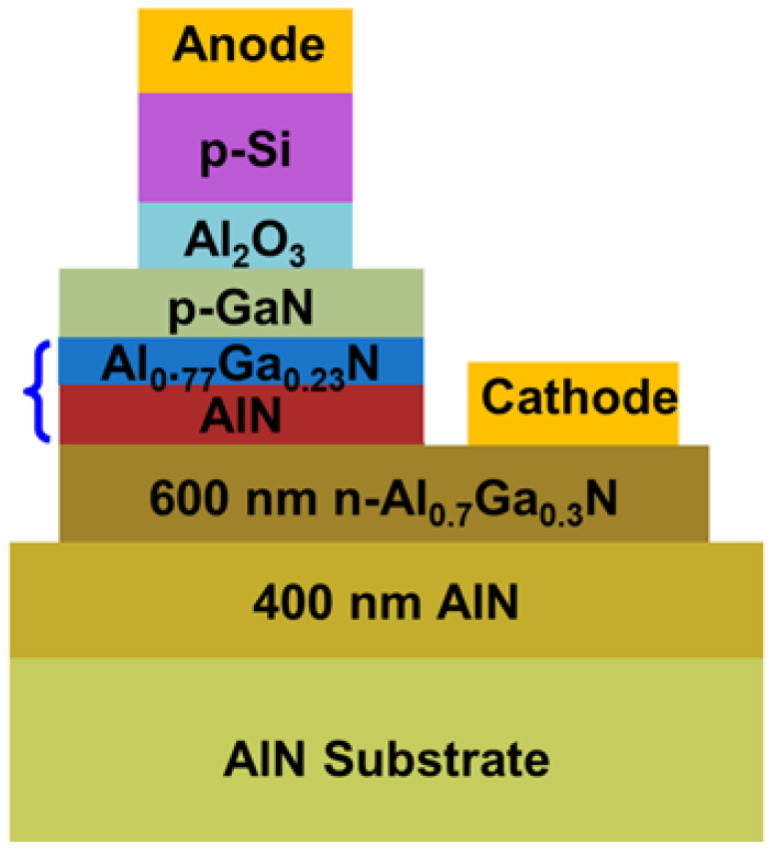
UV-LED fabrication process illustration. An optical microscopic image of fabricated LED. [17] Copyright 2018, AIP Publishing.

Liu et al. worked on a 229 nm AlN/Al_0.77_Ga_0.23_N LED with a p-type Si nanomembrane as both a p-contact and a hole injection layer for high aluminum content and their designed fabrication is shown in Figure 5 [17]. Their structure proved that, for the practical implementation of UVC LEDs, the UVC emission from electrically injected diode structures enabled by p-type Si nano-membrane hole injection layers could provide a route to diode lasers in the future.

S. H. Lin examined the concept of a reflective passivation layer (RPL) consisting of HfO_2_/SiO_2_ stacks as distributed Bragg reflectors deposited on DUV-LEDs with different *p*-GaN thicknesses [18]. This RPL-based scheme enhanced the EQE droops of the deep UV-LEDs with *p*-GaN layers, causing an increase in *P_out_* by 18.4% and 39.4% at the injection current of 500 mA and by 17.9% and 37.9% for 1000 mA, respectively. With the thick *p*-GaN, the efficiency droops with and without the RPL were 20.1% and 19.1% while it was 18.0% and 15.6% with and without RPL, respectively, for thin *p*-GaN. B. T. Tran et al. fabricated a high-quality AlN template on 2 inch micro-circle patterned Si substrate [19]. They reported the lowest dislocation density of 107 cm^−2^ for Si substrate. They also observed a strong electroluminescence peak for an AlGaN-based deep UV-LED, which is useful for developing highly efficient deep UV-LEDs.

Y. Kashima et al. improved the light-extraction efficiency (LEE) by introducing a highly reflective photonic crystal (HR-PhC) into the p-AlGaN contact layer of AlGaN-based deep UV-LEDs, and hence achieved a better EQE [20]. This HR-PhC was fabricated using nanoimprinting and dry etching and a reflective Ni/Mg p-type electrode was deposited on the HR-PhC layer. The external quantum efficiency was increased from 4.8 to 10% by introducing this above mentioned fabrication technique.

**Figure 6:**
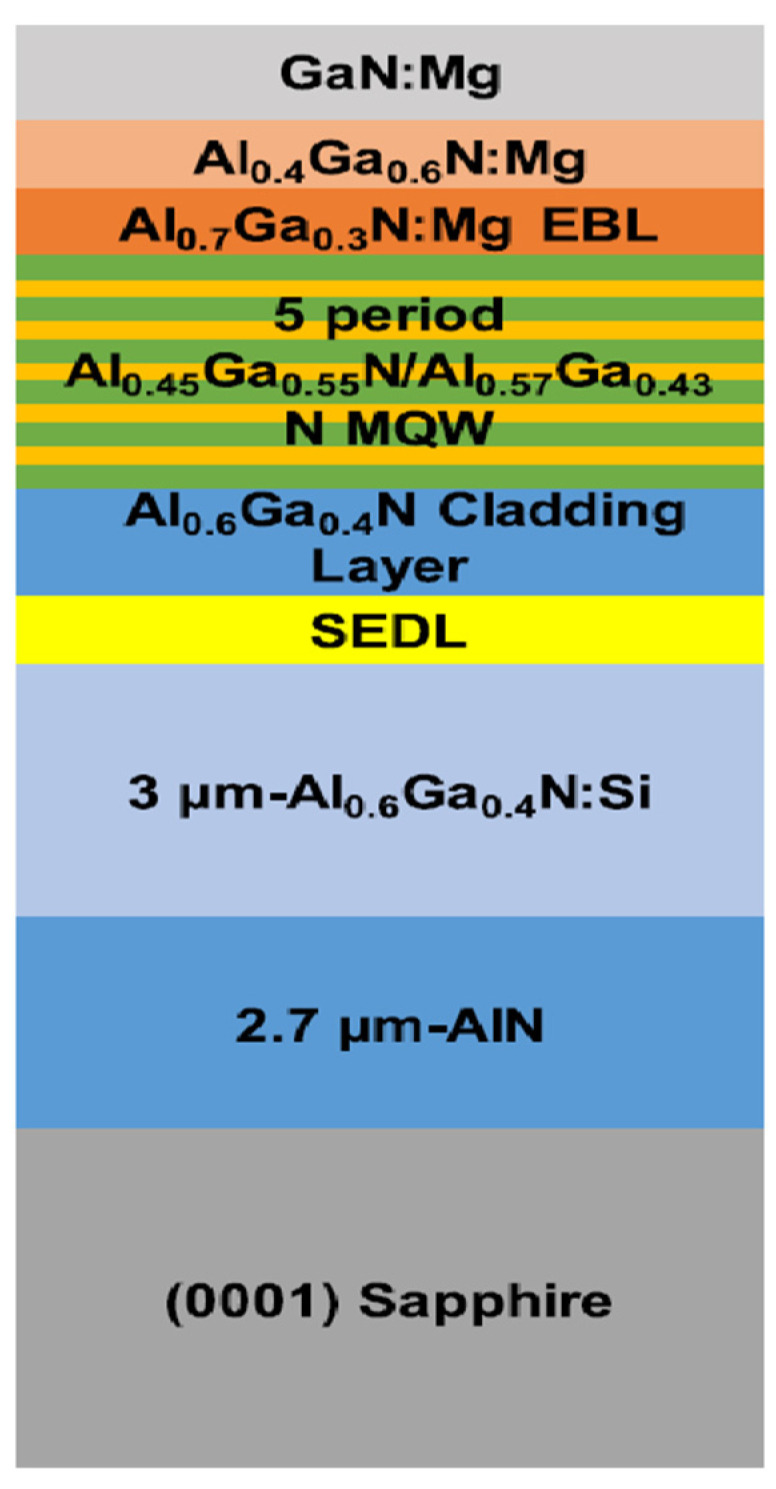
A schematic of DUV-LED structure with chirped SEDL. [23] Copyright 2018, AIP Publishing.

Z. H. Zhang et al. specifically designed DUV-LED with a superlattice p-type electron blocking layer (p-EBL). This superlattice p-EBL enabled a high hole concentration, which in turn increased the hole injection efficiency into the MQWs [21]. K. Nagamatsu et al. investigated the emission intensity of AlGaN-based deep-UV-LEDs under the effect of a highly Mg-doped strain relaxation layer [22]. They concluded that due to excessive Mg doping, the self-formed voids are formed which were very influential in increasing strain relaxation and resulted in improved output efficiency. Furthermore, they found that with a strain relaxation layer on sputter-annealed AlN, the emission intensity of LEDs increased 11-fold compared to the absence of strain relaxation layer. Jiahui Hu proposed a superlattice electron deceleration layer (SEDL) to slow down the electrons injected to the active region, thereby improving the radiative recombination, as shown in Figure 6. External quantum efficiency of 3.43% was calculated at 40 mA, indicating that the WPE is 2.41% with Al-content chirped SEDL [23].

**Figure 7:**
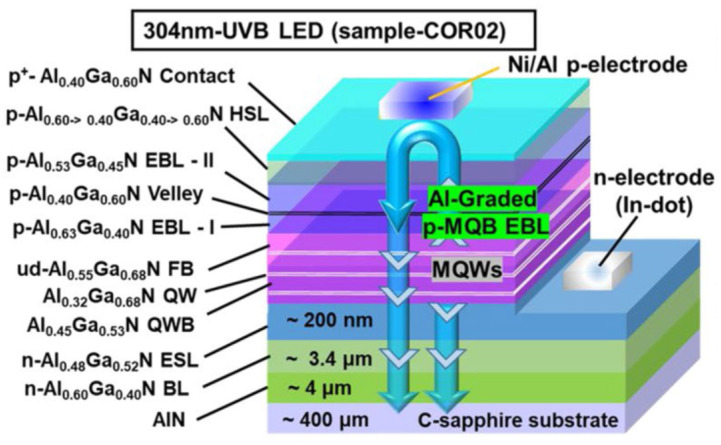
Schematic diagram of 304 nm-band AlGaN-based UVB-LED. [24] Copyright 2022, Springer Nature Publishing.

Muhammad Ajmal et al. investigated the effect of Al-graded p-type MQB EBL and Al-graded p-AlGaN hole source layer (HSL) on the production and injection of 3D holes in the active region [24]. Their design provided a substantial improvement in the efficiency and light output power of about 8.2% and 36 mW respectively. Even in the absence of standard package, they observed an improved efficiency of up to 9.6% and *P_out_* of 40 mW by the structure as shown in Figure 7. The different manufacturing techniques and their outcomes are compared in Table 1.

## 3. Degradation Due to Current Stress

Reduced optical power has been found dependent on constant current operation as the higher the current density, the faster the degradation [36]. For visible LEDs, based on nitride materials, a current driven degradation has been observed [37], and the lifetime of white LEDs was found to be inversely proportional to the current with an exponent of 0.17 [38]. Even a small amount of current has been observed to cause the degradation of GaN-based LED chips [3,39,40,41,42,43]. The degradation is usually attributed to the production of nonradiative recombination centers in the active layer [44,45]. Several mechanisms have been reviewed here to understand better how to work out the possible reasons and ways to increase the reliability in the following part of this section.

**Figure 8:**
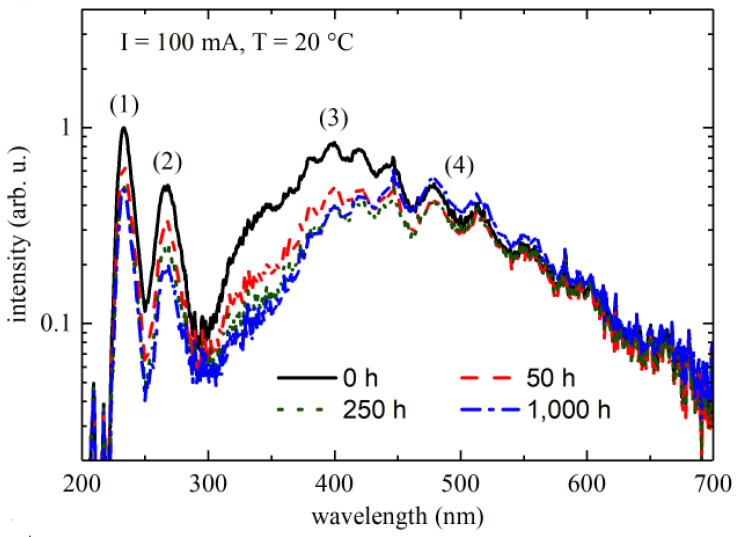
Emission spectra for different operating taken at 20 °C and 100 mA. Reproduced with permission. [36] Copyright 2018, IOP Publishing.

Johannes Glaab et al. employed multiple-quantum-well (MQW) DUV-LEDs pouring out 233 nm wavelengths stressed at a persistent current of 100 mA for 1000 h of processing, as indicated by Figure 8 [36]. In the first 250 h, the MQW luminescence was lowered, while for the rest of the processing time, the emission power of MQW was observed to be stable. In comparison to high-current regimes, the lower ones indicated the rigorous change in the emission power when observed with changing drive current. As the formerly mentioned trend, all these effects were remarkable for the first 250 h.

**Figure 9:**
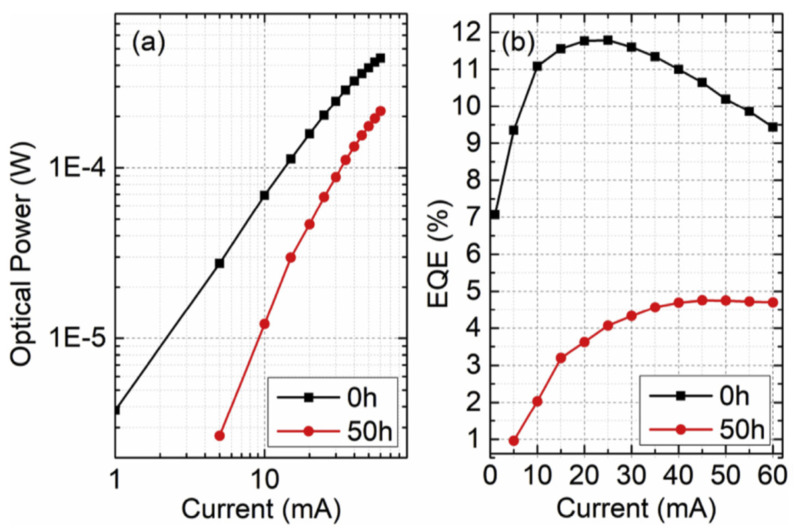
(**a**) Optical power and (**b**) EQE dependence on the forward current for LED B before and after 50 h aging. Reproduced with permission. [47] Copyright 2019, Elsevier.

Craig G. Moe et al. employed single-chip packaged LEDs (emitting 285 nm) to measure the lifespan under the influence of persistent current inoculation at 20 and 75 mA [46]. In comparison to electrically stressed devices, the thermally stressed indicated low-grade degradation. Huixin Xiu et al. explained that the optical degradation caused by the leakage current was potentially the significant degradation mode of UV-LEDs [47]. Their results showed that the partial interaction of the contact metals with p-type materials boost up the demeaning of LEDs. The optical power (OP) versus (L-I) curves, i.e., forwarding current for 50 h aging are illustrated in Figure 9a. The slope indicates the ruling position of nonradiative recombination of Shockley-Read-Hall (RSH), succeeding the 50 h aging, the RSH nonradiative recombination rises, suggesting excessive imperfections. External quantum efficiency (EQE) about aging for LED is indicated too in Figure 9b.

**Figure 10:**
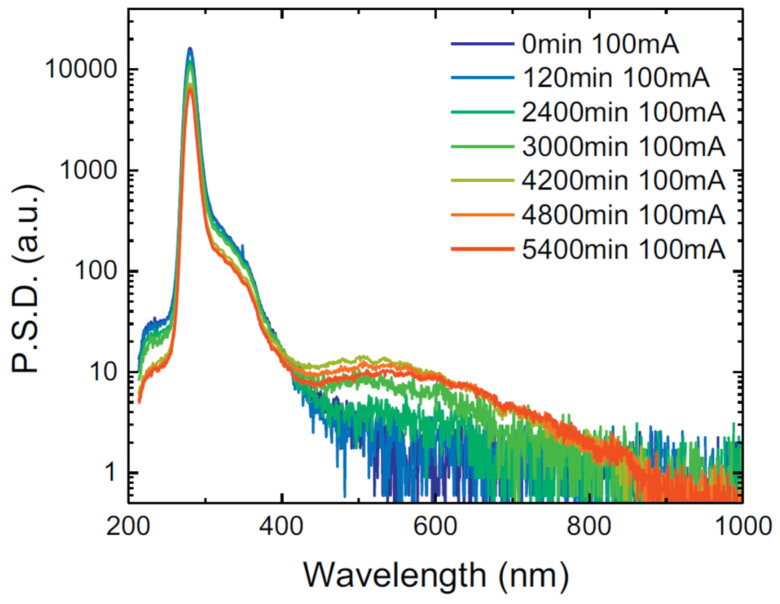
Spectrum variation during 500 mA stress test. Reproduced with permission. [48] Copyright 2018, Elsevier.

N. Trivellin et al. reported on the spectral characterization with temperature variations and studied the reliability of DUV-LEDs. For both stress currents of 350 mA and 500 mA, continuous current reliability testing was carried out. The results indicated a subsequent degradation of the 280 and 354 nm emission peaks (Figure 10). Corresponding to the stress applied, a novel emission peak emerged: a relatively wide peak with its maxima located at 540 nm approximately [48].

Pinos et al. studied the emission spectrum from a 285 nm AlGaN quantum well light-emitting diode (QW-LED). The results indicated that certain regions (which are micrometer-sized, domain-like areas) having lower AlN molar fractions pour out light with higher intensity. Furthermore, the various experiments performed revealed that intensity from these regions raised with time and a redshift in wavelength is observed [49]. In another study, Pinos et al. described the degradation under the influence of elevated current stress of AlGaN quantum well-based LEDs pouring out light of 285 and 310 nm wavelength. The results revealed that elevated concentration of nitrogen-vacancy and tunneling current as well as partial reimbursement of the p-doping caused to decrease in the emission intensity during the aging process [50]. Jan Ruschel et al. investigated the degradation in UV-LEDs for 1000 h of stress [51]. The influence of operation current within the range 50–300 mA was also studied in comparison to current densities in the particular range from 34 A/cm^2^ to 201 A/cm^2^. In aging operations, the optical power reduction was robustly speed-up for higher current [52,53].

Matteo Meneghini et al. explored DUV-LEDs and analyzed the reliability at a DC stress of 20 mA. In response to aging, the devices intimated a lesser optical power [39]. For minor levels of current, the decrease in OP suggested that the stress resulted in excessive paths for nonradiative recombination and appearance of defects. In another study, they concluded that: (1) persistent current stress elevated the nonradiative recombination rate within the active region and consequently the efficiency of GaN-based LEDs was lowered [3]. They also explored 310 nm UV-LEDs and found that the time dependency of optical power (OP) could be controlled by a scaling factor which is given by the cube of the operating current density. This indicated the greater influence of current density on the lifespan of such devices. In order to improve the reliability, they strongly recommended to enhance the active area [54].

**Figure 11:**
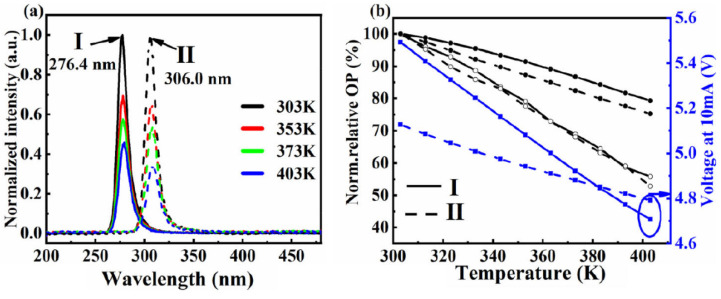
(**a**) The normalized EL spectra of 276 nm and 306 nm LED at current of 0.5 mA. (**b**) The normalized OP at various temperatures. [55] Copyright 2022, AIP.

We also studied the reliability of 276 nm and 306 nm AlGaN-based UV-LEDs for the temperature range of 303–403 K [55]. Figure 11a shows the EL spectra of two LEDs as a function of temperature for the given range. The two UV-LEDs peaked at 276.4 nm and 306.0 nm at 303 K. The increasing temperature caused a decrease in EL intensity of these LEDs. The Figure 11b shows how the luminous efficiency is affected by operating temperature for different current levels. The optical power dropped to 54.2% and 55.2% for 276 nm and 306 nm LEDs, respectively. We found that nonradiative recombination occurs at low currents when carriers tunnel to the space charge region.

**Figure 12:**
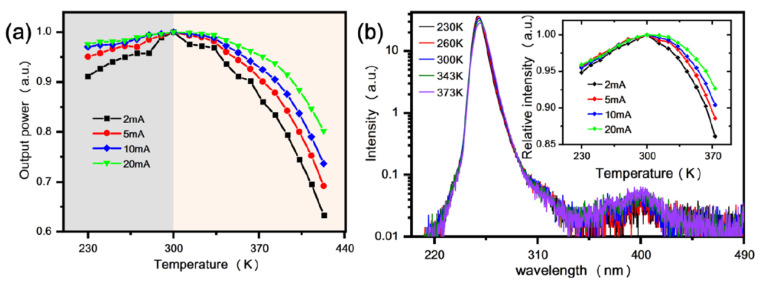
(**a**) Normalized OP of UV-C LED with temperature. (**b**) The EL spectra of LED at different temperatures measured at 2 mA. [56] Copyright 2022, Springer. Finally, we have summarized the operating conditions and related output as given in Table 1.

Very recently, we explored the reliability of 255 nm UVC LEDs [56]. We unveiled that when thermal defects are activated there exists the trap assisted tunneling that causes intensity reduction at elevated temperature. It was also found that the optical power changes emerge at 300 K while at increasing temperature range the optical power decreases (Figure 12a), but for the temperature below 300 K, till 230 K, the optical power increases with temperature enhancement, so it was concluded that the optical power degradation mechanisms is very sensitive for the operating temperature below and above 300 K (Figure 12b). The Table 2 summarizes the different current stress conditions and related degradation details.

## 4. Degradation Due to Defect Creation

The existence of defects in optoelectronic devices are usually investigated using photocurrent (PC) spectroscopy [58]. This strategy works by exciting carriers present in QWs using the light with a specific wavelength and the relative disparity in the PC signal is then estimated. For ideal LEDs, if the photonic energy is lesser than the bandgap, there would be no PC because carriers will not be excited in this case. With the escalation of photonic energy for deeper energy levels, more electrons are excited and hence it launches more carriers. Devices with increased Al content have nitrogen vacancies (N-vacancies) in the emission band because the cladding bears the optical transitions. In the aging process, the appearance of the emission band suggests that the N-vacancies play an important role. It gives rise to defects and increasing conductivity channels. Also, local heating and current crowding are observed due to excessive defects because these act as nonradiative recombination centers [59]. All this gives rise to not only the swift aging of the device but also the low quantum efficiency [49].

Excessive defects, lead to greater degradation which is evident from lowering optical power at the minor levels of current. This shows that the defects in the active layer increase with raising stress. Substrates with large lattice mismatches provide the site for heteroepitaxial growth, as a result of this mismatch, excessive defects and dislocation densities appear. In order to achieve an elevated performance rate, cautious processing and optimization of the growth are required [10]. Based on the literature, in LEDs, optical degradation is described as the rise of defects concentration in the active layer of the devices. The concentration of defects in the active layer is stipulated by the intensity of the emission bands. To acquire a monochromatic and high-quality UV emission, the optimization of structural parameters and growth conditions is necessary [54].

Desiree Monti et al. employed deep-level transient spectroscopy (DLTS) and photocurrent (PC) spectroscopy to describe the behavior of defects in modifying the electro-optical performance of devices [58]. They demonstrated that optical degradation corresponded with the appearance of defects generated at about 2.5 eV under the conduction band (CB) edge. This defect concentration was assumed to play a part in trap-assisted tunneling (TAT) processes and Shockley–Read–Hall (SRH) recombination. From DLTS analysis, they concluded that a positive maximum was associated with the trap levels of minority carriers, whereas for the majority carriers a negative peak was observed [60]. Their DLTS analysis showed that the active region occupied Mg-related acceptor traps, and by changing their concentration the p-type region and the ohmic contacts showed a modification in electrical characteristics. A.Pinos et al. studied the QW emission and found an EL peak in the 270 nm which had low energy [61]. Their finding suggested that the cladding layers of virgin devices contained N vacancies. These vacancies caused crucial LED aging as they seed the generation of defects and high conductivity channels.

**Figure 13:**
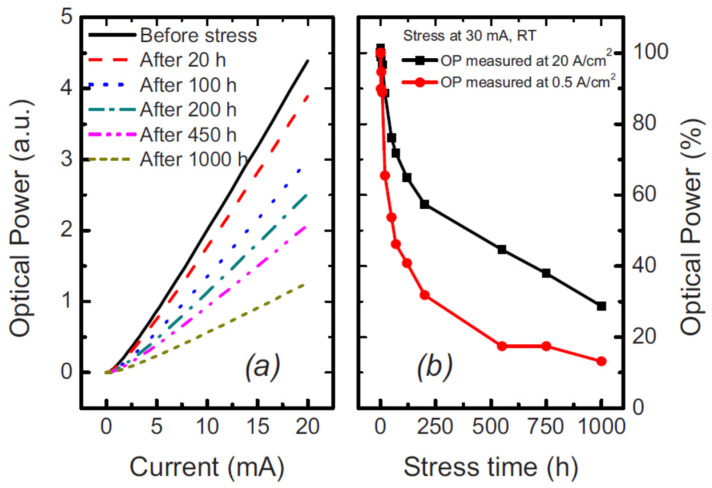
(**a**) L-I characteristics, (**b**) OP degradation. Reproduced with permission. [62] Copyright 2010, AIP.

Matteo Meneghini et al. described that at lesser current levels, the optical power (OP) lowering was outstanding which suggested that excessive defects favored the degradation [62]. Their work indicated that the increase of the defectiveness in the active layer led to greater degradation by gradually lowering the radiative efficiency (Figure 13). Their investigations indicated that the stress elevates the defect concentration in the active layer, along with the further lowering of the radiative efficiency.

**Figure 14:**
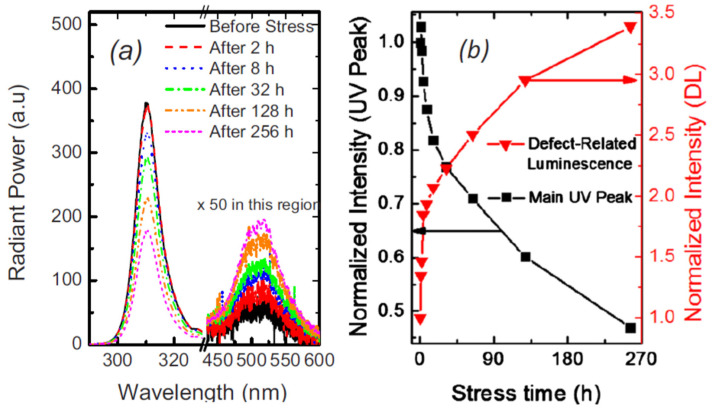
(**a**) EL spectra with stress at 17 A/cm^2^, RT. (**b**) Variation in the intensity of peaks under stress. Reproduced with permission. [62] Copyright 2010, AIP.

As shown in Figure 14, the main UV emission peak showed a reduction as a consequence of stress. The above investigations indicated that stress caused an increase in the defects concentration which is the source of yellow luminescence in the AlGaN-based LEDs.

**Figure 15:**
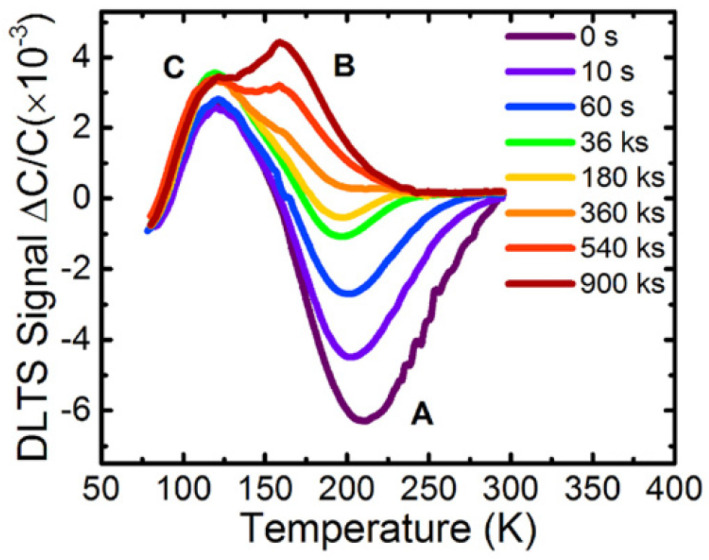
DLTS spectra of UV-C LED measured under a reverse bias of −6 V. Reproduced with permission. [64] Copyright 20120, AIP.

In another study, M. Menghini et al. explained that the optical degradation was more remarkable for lesser levels of current: this outcome indicated a rise in the defect concentration in the active layer [63], which caused degradation. Ying Zhe Wang et al. investigated the role of defects in the degradation of AlGaN-based UV-C light-emitting diodes (LEDs) under the influence of non-varying current stress [64]. They used deep-level transient spectroscopy (DLTS) to investigate underlying defect evolution. The Figure 15 showed that a peak at about 120 K remained constant while under stress [65]. A shoulder emerged at 160 K and then grew up, suggesting the correlation between the generated defects.

D Monti et al. analyzed the degradation in (In)AlGaN-based UV-B LEDs and observed an insignificant emission wavelength of 310 nm, yielded to consistent current stress at an elevated current density of 350 A/cm^2^ [66]. After passing 50 h, they noticed that the current lower than the turn-on voltage at *V* = 2 V raised as a square root of time dependence. The occurrence of the diffusion process was evident from this behavior. It gave rise to point defects which in turn led to the rise of nonradiative recombination in the LED. F. Piva et al. worked out the optical, spectral, electrical, and steady-state photo-capacitance (SSPC) analysis during stress and illustrated the existence of two distinct degradation processes [67]. In the first 1000 min of stress, the first process occurred which was evident by the lowering of injection efficiency. This was attributed to the defect generation dynamics corresponding to the de-hydrogenation of gallium vacancies. After passing 1000 min of stress, the second process started. This corresponded with the appearance of mid-gap defects, which further led to the rise in nonradiative recombination via trap-assisted phenomenon [68]. As a result, a remarkable increase in the current was observed.

Nevertheless, the degradation of AlGaN-based UV-C LEDs is not still fully explored in terms of defects: generation and location of defects, behaviors of defects, etc. Hence, undoubtedly there is a desperate need for a detailed investigation and an in-depth analysis of the nature of defects. This will add to the device’s reliability by improving the fabrication process of the device fabrication process [64].

## 5. Degradation Effects on C–V Characteristics

In order to obtain distinct and accurate information of fixing the free charge in the active layer of the heterostructure, the apparent charge distribution (ACD) profiles are generally use the capacitance–voltage (C-V) measurements [62,69,70]. From the capacitance–voltage (C-V) measurements, it is possible to bring out the broadness of the space-charge region (SCR) and the quantity of the charge that depends upon the voltage at the junction, so that the capacitance of the barrier can be modified by altering given voltage. From theoretical investigations, it was inferred that the concentration of the C-V measurement was roughly equivalent to the aggregation of free carriers. Also, it confirms the charge preservation in heterojunction materials. Thus, it was inferred that in the active region, the relevant carrier distribution was supposed to be the n-side doping [42,71]. It is worth bearing in mind that the same supposition was used as an estimation in the extraction of the clear depths and carrier concentration. The SCR often expands on both sides because the p-side contains limited charge concentration [72].

**Figure 16:**
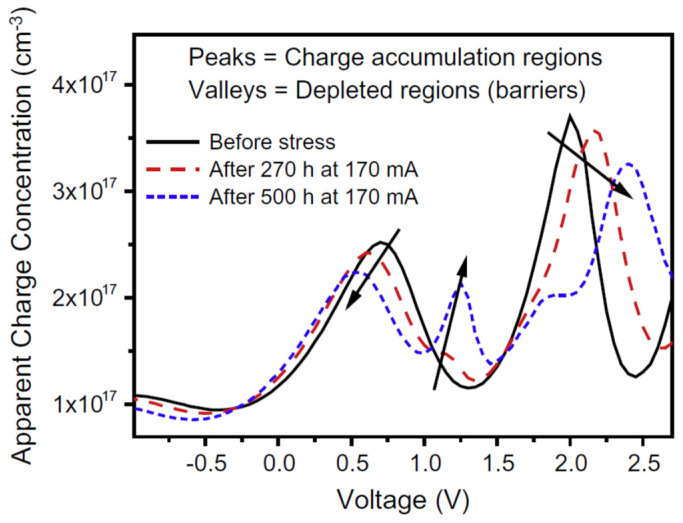
ACD profiles measured before and during stress at 170 mA. Reproduced with permission. [54] Copyright 2010, Elsevier.

M. Meneghini et al. used capacitance–voltage (C-V) characterization to investigate the workable modification in the charge distribution and dopants within the active layer generated by the stress treatment [54]. In Figure 16 they illustrated the apparent charge distribution (ACD) profiles brought about one of the tested specimens throughout the stress analysis. Their outcome corresponded to the generation as well as the dispersion of charged centers within the QW region. It also brings forth information on the positioning of the semiconductor region modified by the demeaning procedure [42].

**Figure 17:**
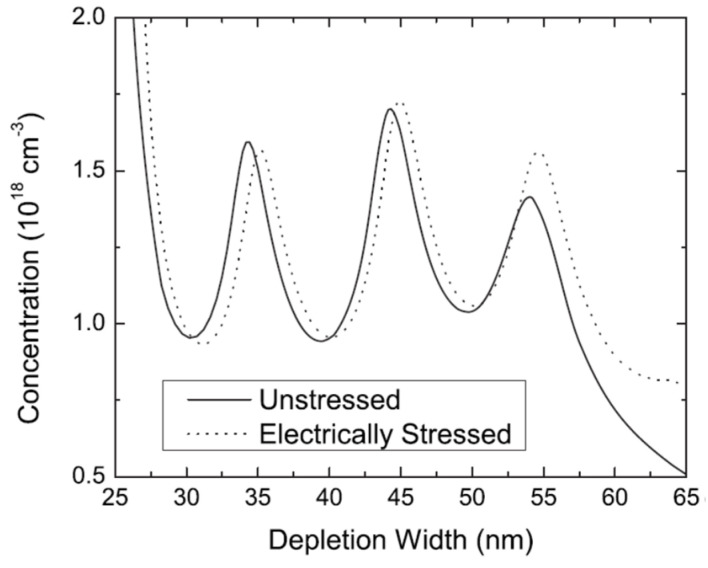
ACD profile of an unstressed and electrically stressed device as a function of depletion width. Reproduced with permission. [62] Copyright 2010, AIP.

Matteo Meneghini et al. in another study obtained the positioning of the degraded area using capacitance–voltage (C-V) measurement. They found that the stress caused the free carrier density to reduce in the active region and modified the distribution of free charge close to the QW. Hence, as a result of stress, the active region charge could be reimbursed to a certain extent [62]. Craig G. Moe et al. performed capacitance–voltage (C-V) measurements not only on the unstressed devices but also on the electrically stressed devices at 20 mA. The electrically stressed QWs were positioned at a larger obvious depletion width as compared to the unstressed device [46]. When the *p*-side to the biased QW was observed, a broader depletion width was noticed. This conclusion indicated that the depletion region expanded into the *p*-layer right after the electrical degradation took place. The possible reason is that near the heterojunction, the *p*-type carriers in the barrier region got reimbursed (Figure 17).

**Figure 18:**
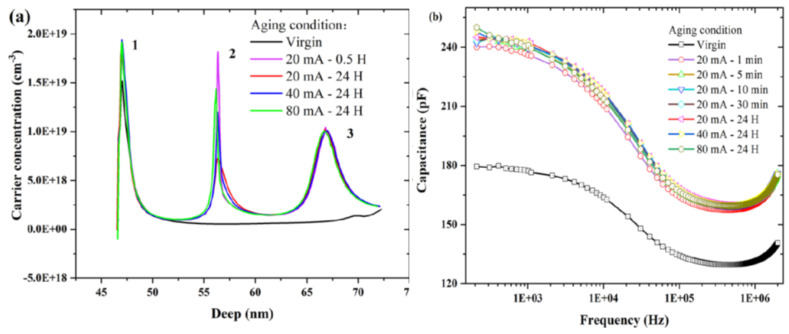
(**a**) ACD profiles of stressed and unstressed devices. (**b**) The capacitance vs. frequency diagram. Reproduced with permission. [73] Copyright 2021, AIP.

Our group figured out the CV measurements in a frequency range of 200 Hz–2 MHz to verify the subordination of the capacitance characteristics [73]. The capacitance–frequency is illustrated in Figure 18. It is clear that the application of the current in the first minute accumulated a large increment in the capacitance. Later on, the capacitance was stabilized with minute variations even when the device was stressed consistently. In the first 0.5 h of degradation, we noticed the carrier variations within the range of 200 Hz-2 MHz for a minute’s stress. Hence, we concluded that the consistent longer reduction of optical power (OP) is not probable to be activated by the rise in the traps [74].

**Figure 19:**
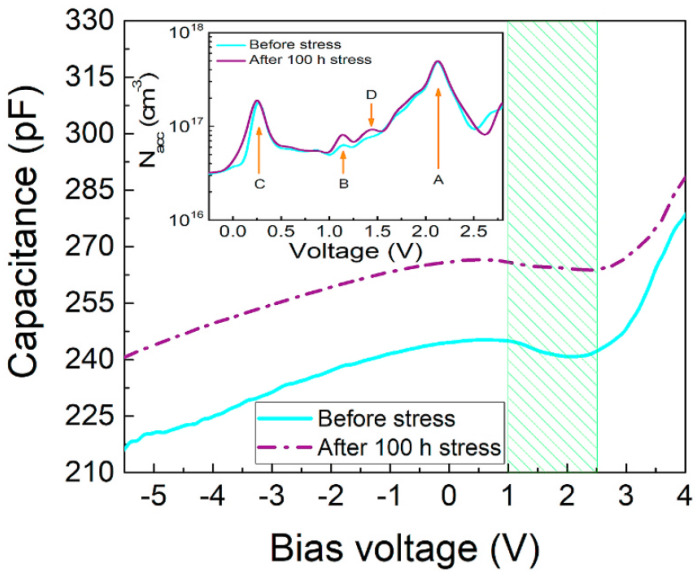
Typical C-V curves of the investigated DUV LED measured before and after stress treatment. Inset: [75]. Copyright 2020, Elsevier.

In another study, our group carried out the C-V calculations at 1 MHz to observe the device aging [72]. In the forward-bias range (0–5) V, we observed the lowering of capacitance with the rise of the aging current. This observation corresponded to the fact that the capacitance depends on the voltage which in turn was a role of the depletion width (WD) of the p–n junction. As the stress currents raised, the maximum capacitance lowered. This showed that the unstressed devices have lesser depletion widths than stressed one [36]. Using C-V measurements, the ACD profiles of virgin and stressed devices were drawn out to directly vary with the depletion depth, near the frequency of 1 MHz. As shown in Figure 12b, the carrier concentrations in QW II and QW III presented a remarkable increment; however, the LEDs were aged with various currents. Matteo Meneghini et al. also investigated the C-V profile to correlate the degradation process and modified properties of the active region [39]. The charge concentration was elevated by DC-bias aging. During the first 250 h of processing, these changes occurred just like the lowering of optical power. These results indicated that the lowering of optical power and modified charge profiles are consistent with each other and it was in accordance with already published work on InGaN-based blue LEDs [42]. To see the impact of constant current stress on the device capacitance, Pradip Dalapati et al. determined C-V characteristics about the stress injection, which is illustrated in Figure 14. The capacitance as a whole raised in both forward and reverse bias regions. Thus, as a result of 100 h current stress, the net modified charge-concentration was changed entirely within the active region [75]. In addition, the increase of capacitance could be attributed to the depletion width narrowing [76,77]. The narrowing of depletion width get around to the powerful internal electric field inside the LED and the large tunneling current [76]. As it is evident in Figure 16, the magnitude of the negative slope was decreased after the stress treatment, indicating the strong effect of degradation governed within the second SCR. To get a clear scenario of the charge distribution, they had extrapolated the apparent charge distribution (ACD) profiles from the C-V measurements which are shown in the inset of Figure 19.

Zhanhong Ma, et al. also performed the C-V depiction of the devices while carrying out the stress treatment [78]. When the LED was set off under reverse bias voltage, the whole capacitance was dominated by the barrier capacitance. However, when the LED was under forwarding bias, the major part was attributed to the diffusion capacitance. With the fall of the reverse bias voltage, there was a rise in the capacitance of the micro-LED. This suggested that the borderline of the region containing the space charge has moved [45,79].

**Figure 20:**
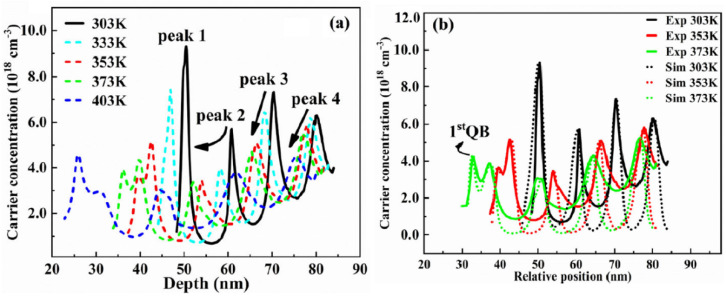
(**a**) The ACD profiles of 276 nm LED at different temperatures, obtained from CV measurements at 1 MHz versus depletion depth (**b**) 1D simulation and experimental data of ACD profiles. [55] Copyright 2022, Springer.

One of our recent explorations of ACD profile is depicted in Figure 20a,b [55]. It was observed that the increasing temperatures causes a reduction in peak height, but also increases its width. To strengthen our findings, we also performed the numerical simulation on the ACD using SilenSe software and set the temperature range as 303–403 K. The simultaneous results of experiment and our simulation are shown in Figure 20b.

**Figure 21:**
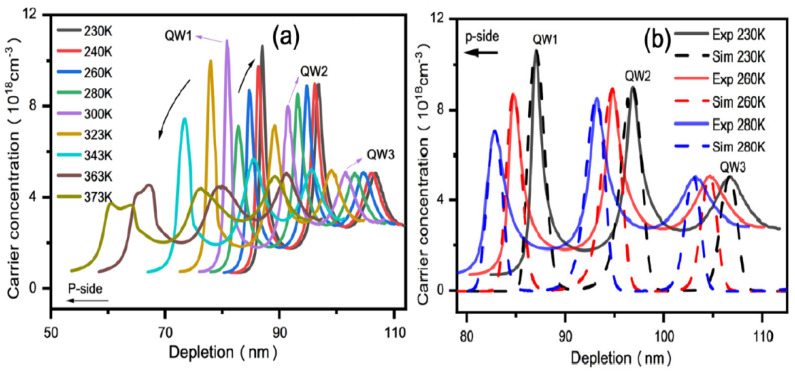
(**a**) The ACD profile of 255 nm LED at different temperatures; (**b**) 1D simulation of ACD profile of 255 nm LED at different temperatures. [56] Copyright 2022, Springer.

Our group also performed CV measurements on 255 nm UV-LEDs and the obtained ACD function is shown in Figure 21a [56]. It can be seen that the increase in temperature caused a decrease in carrier peak of the first quantum well rapidly, so we concluded that at elevated temperatures, the concentration of carriers in the well decrease rapidly and these carriers also diffuse to the p-side EBL at 373 K. For support of our findings, we also used Silense 1D simulator to observe the carrier distribution during thermal changes as shown in Figure 21b. Finally, we have summarized the operating conditions and corresponding capacitance response in Table 3.

## 6. Degradation Effects on I–V Characteristics

For the degradation analysis using I-V curves, three main regions have been discussed mostly in the literature: (1) the reverse bias region; (2) the low forward-bias region; and (3) the above turn-on voltage bias region. These regions behaved differently when the degradation goes on, as discussed in the following section.

**Figure 22:**
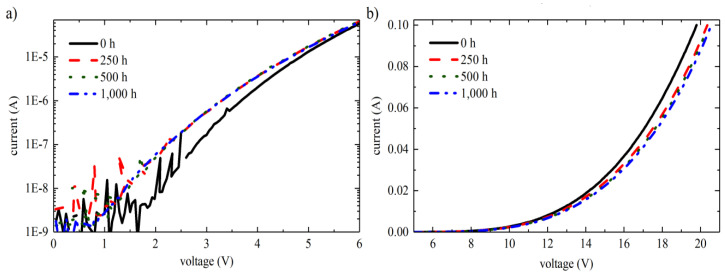
Current-voltage characteristics (**a**) below and (**b**) above the turn-on voltage. Reproduced with permission. [36] Copyright 2018, IOP Publishing.

Figure 22a,b illustrates the forward-bias I-V characteristic curve of one LED carried out after a distinct procedure interval by Johannes Glaab et al. The current for a corresponding voltage lower than the start-up voltage (_Vturn-on_ = 5.3 V) raised minutely in the interval of initial 250 h of the procedure (see Figure 22a) [36]. For operation times greater than 250 h, the current for this relevant voltage holds out consistently. In contrast to that, the current at a rooted voltage greater than the start-up voltage lowered consistently (Figure 22b).

**Figure 23:**
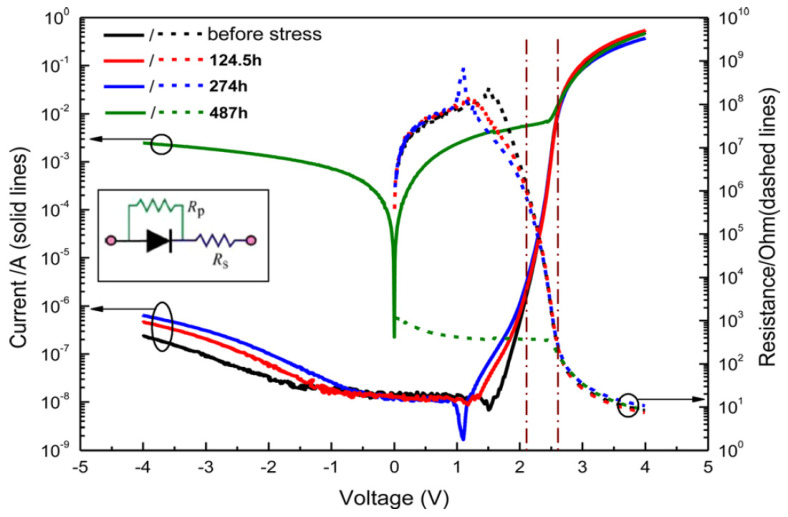
I-V curves and resistance characteristics of an LED: before stress, and 124.5, 274, and 487 h after stress. Reproduced with permission. [57] Copyright 2012, AIP.

Lilin Liu et al. extracted the I-V curves and resistance traits of an LED chip before as well as after stress application at 60 A/cm^2^ and 60 °C for 124.5, 274, and 487 h respectively, as illustrated by Figure 23 [57]. The forward portion of a typical I-V characteristic curve was split up into three regions: the tunneling current region (I, the bias <2.1 V) corresponded to the production of the defect and the activation of the dopant, the diffusion-recombination current region, and the series resistance region [80].

Forward I-V measurements of Menighini et al. indicated that the stress did not indicate the modification in the operating voltage of the devices, which suggests consistency of ohmic contact and resistivity of neutral regions throughout the stress time. Conversely, they noticed an increment in the device in reverse current and lower of forwarding bias region [40]. In another study, they proposed a thorough investigation of the I-V characteristics curves of the specimens and explained that dc stress can also cause a rise in the reverse and low-forward bias (*V <* 2 V) current components: this outcome holds up the notion that stress can stimulate the dispersion of imperfections in the active areas of the specimens [3].

**Figure 24:**
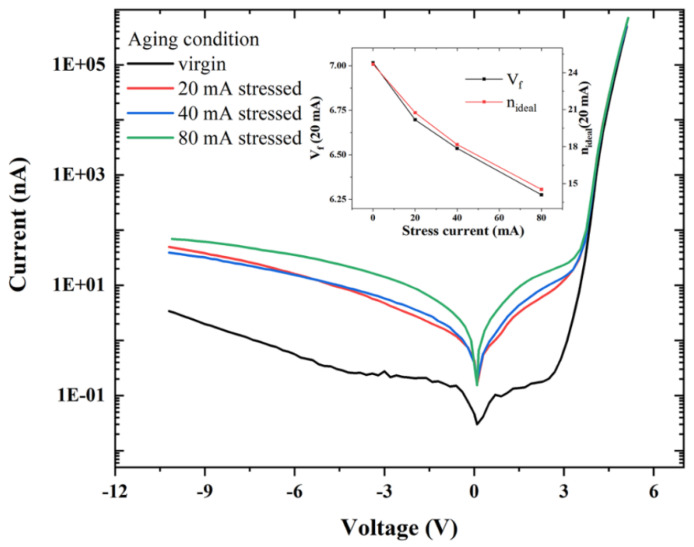
The I–V characteristics of samples stressed at different stress currents. Inset: forward voltage (V_f_) and ideality factor variation (n_ideal_) under different stress currents. Reproduced with permission [50]. Copyright 2021, AIP.

Our group also worked out the I–V characteristic curves of the device with various stressing currents for 24 h [73]. In comparison to the unstressed device, the rise in the reverse leakage current was observed by 1–2 orders of magnitude; in general, both in the reverse bias region as well as the forward bias region, which was the evidence of higher defect-assisted tunneling (Figure 24) [50].

**Figure 25:**
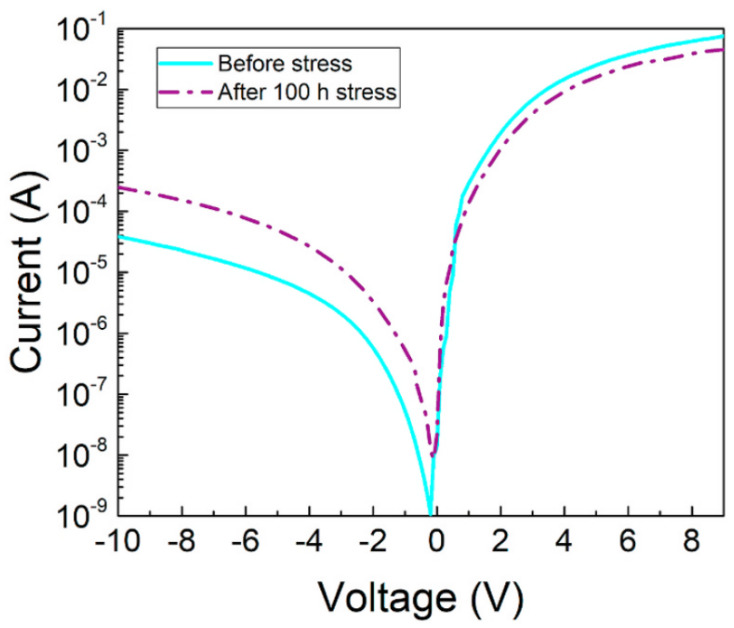
Typical I-V curves of (In)AlGaN-based DUV LED measured before and after stress treatment. Reproduced with permission. [75] Copyright 2020, Elsevier.

Paradip Dalapati et al. figured out that, in a high-forward bias region, the current at a constant voltage lowered remarkably, which transcribed into a rise in drive voltage over 100 h of stress procedure at a consistent current of 60 mA (Figure 25) [75]. 

**Figure 26:**
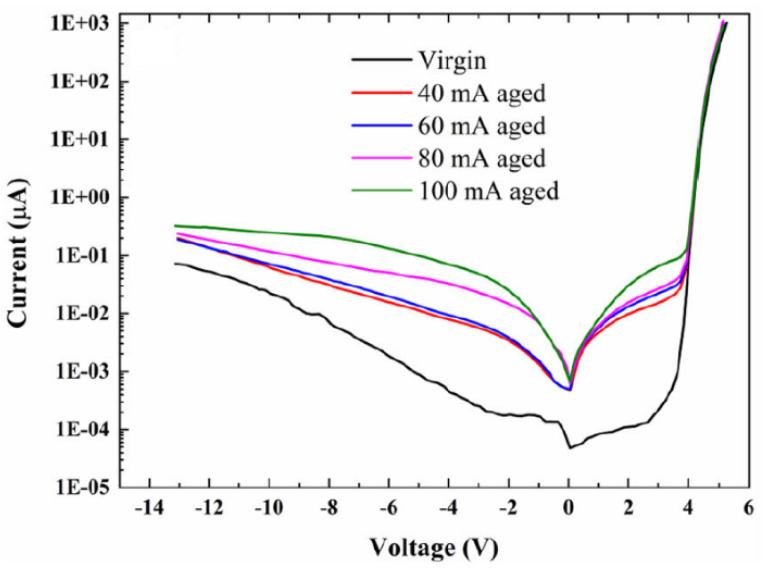
The I–V characteristics of the LEDs stressed at different currents. Reproduced with permission. [72] Copyright 2021, Springer.

In another effort, our group noticed a rise of reverse leakage current and low forward bias region of the electrical stressed devices as shown in Figure 26 [72]. The rise of the reverse-bias leakage current throughout the procedure could be attributed to the increase in defect density about the active region. The forward bias leakage current could be ascribed to the assisted by defect-assisted tunneling procedure causing the degradation of the AlGaN LEDs [64,75].

Zhanhong Ma et al. noticed that with the rise of the time interval of stress, the reverse bias current density of the micro-LED increased up to around five orders of magnitude from 10^−3^ A/cm^2^ to 102 A/cm^2^ at −10 V, and the analogous tunneling current in the lower region of forwarding voltage (<2 V) raised the stress too [78]. This resulted in excessive imperfections within the active area during the degradation [81,82]. Desiree Monti et al. reported the *I*–*V* characteristics curve throughout the device-aging [58]. After a first stable phase (*t* < 0.2 h), the leakage current rose subsequently, ensuing a logarithmic dependence on stress time. The rise in the reverse bias leakage current was attributed to the production of point imperfections about the active region. In the second region (from 0 to ∼4 V), they noticed an increment in the current under the startup voltage. In the high-injection region, they noticed the lowering of the drive voltage in the first 2 h followed by subsequent growth. F Piva et al. reported the I-V measurements worked out constant current stress experiment. Two regions were detected: in the first one, lower than the turn-on voltage (V < 4.5 V), conduction was intervened strongly by defects. Prior to stress treatment, the current was less than 2 nA [67]. For extended stressing time (>1000 min), a remarkable rise in current conduction was detected for 1 V < V < 3 V, i.e., lower than the startup voltage of the PN junction. This result was attributed to the rise in the imperfection density in the active area of the equipment. The I-V characteristic curves were also obtained by Monti et al. about the stress on one of the examined specimens [66]. They were able to identify a rise in the reverse current throughout the stress, which could be attributed to the production of point defects stimulating parasitic-current tracks throughout the active area, and a rise in the carrier production components [83].

**Figure 27:**
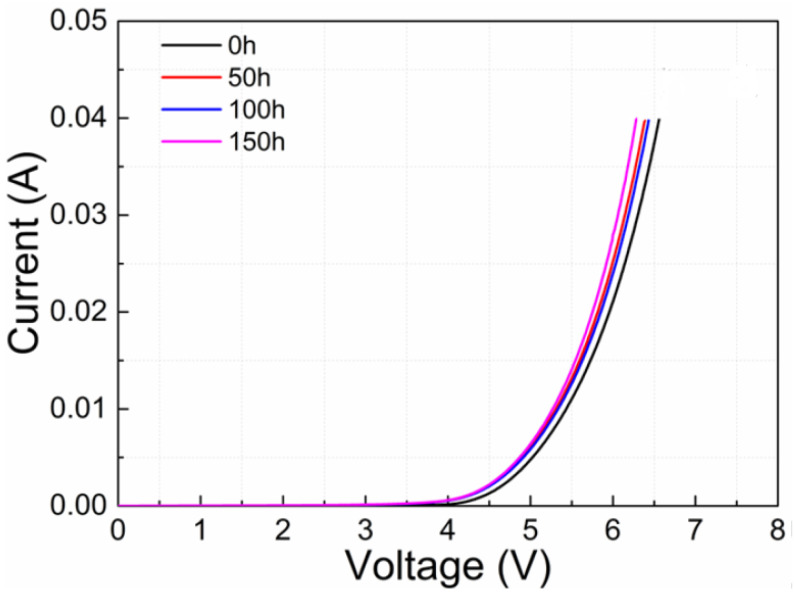
I-V characteristics of the 260 nm ultraviolet LED, measured at different stress times. Reproduced with permission. [81] Copyright 2019, Elsevier.

To achieve a deeper comprehension of the physical mechanism of degradation, Zhanhong Ma et al. investigated the I-V characteristic curves of the LED specimens throughout the aging period, as shown in Figure 27 [81]. In the reverse bias region, the reverse leakage current raised throughout the aging interval. It rose at first and then leakage current stabilized subsequently, succeeding a logarithmic dependence on stress time.

**Figure 28:**
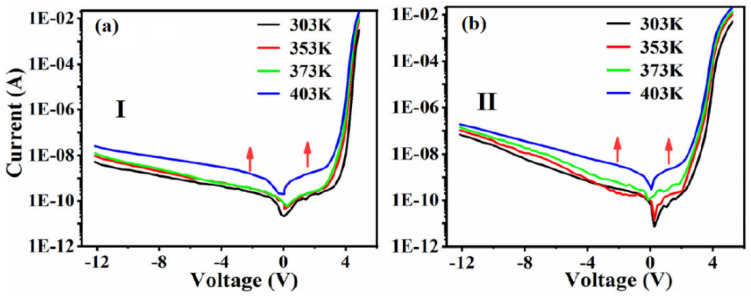
(**a**) The I-V curves of the 276 nm LED and (**b**) 306 nm LED. [56] Copyright 2022, Springer.

In one of our studies, we worked out I-V curves of 276 nm (sample-I) and 306 nm (sample-II) UV-LEDs are shown in Figure 28a,b, which can also be divided into three regions, as mentioned above for such I-V results [56]. For both of the samples, overall currents were enhanced with temperature. On the other hand, in high-conduction voltage region the current was not increased in significant way as compared to other two regions.

**Figure 29:**
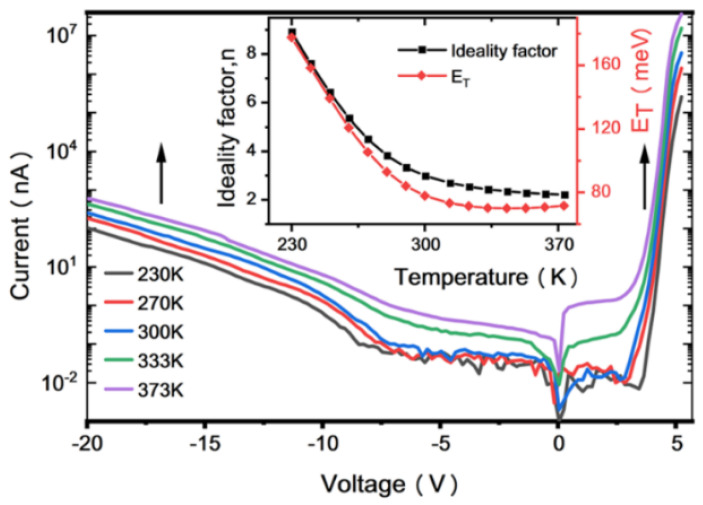
I–V curves of samples at different temperatures. Inset: the relationship between *η* and *E_T_* with temperature. [55] Copyright 2022, AIP.

Recently we have calculated we have used I-V curves to find the ideality factor *η* and characteristic energy level *E_T_* (*E_T_ = ηkT*) [55]. How each of these two parameters behave with the change in temperature is explained in Figure 29, inset.

It can be seen from the figure that characteristic energy level is independent of temperature for elevated temperature above 300 K and trap-assisted tunneling is prevailing, while decrease in temperature causes a reduction in reverse bias current. Table 4 summarize the I-V response to the stress for different LEDS as mentioned in the main text.

## 7. Structural Design Strategies for Better Reliability

**Figure 30:**
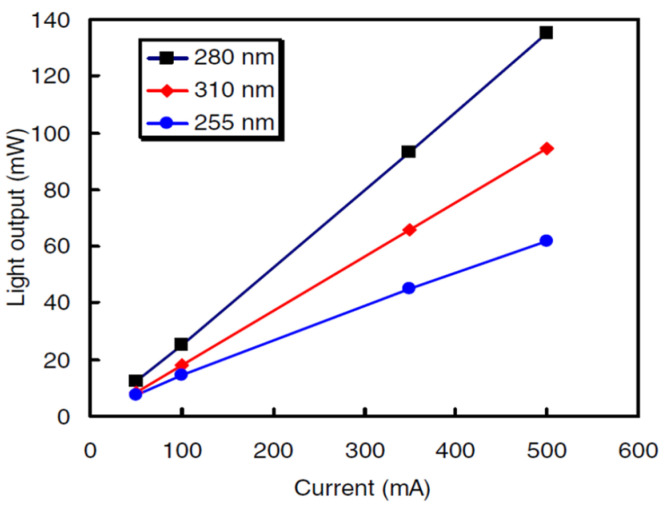
*I–L* characteristics of DUV LEDs operated in pulsed-current mode. Reproduced with permission. [84] Copyright 2014, IOP.

**Figure 31:**
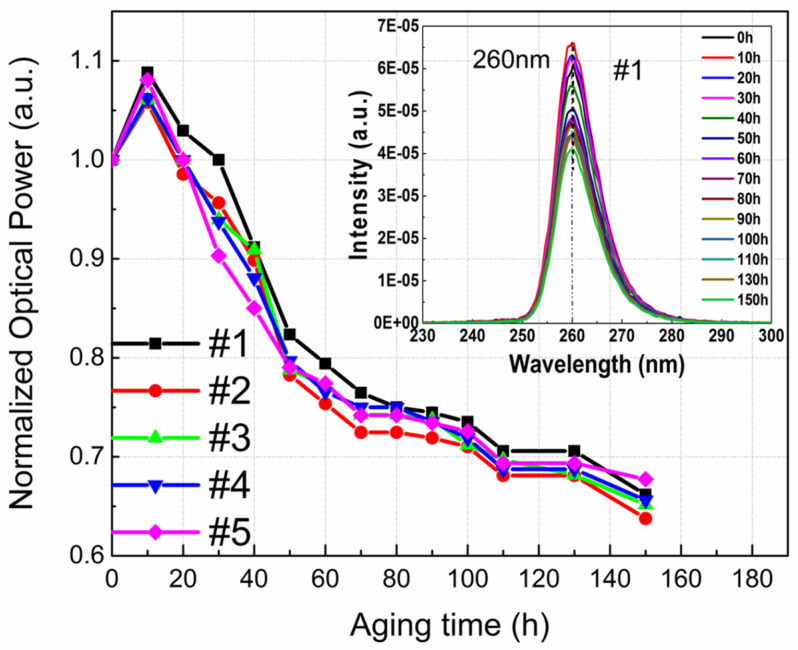
The normalized optical power of the flip-chip AlGaN-based ultraviolet LEDs during aging period. The inset shows the electroluminescence spectra at different stress time. Reproduced with permission. [81] Copyright 2018, Elsevier.

Some research groups worked out constructional developments that led to the enhancement of the reliability of DUV-LEDs. A Fujioka et al. noticed that a lower value current functioning does not often get around to an extended lifespan but designing the chips with a satisfactory size is also necessary [84]. Their 255, 280, and 310 nm LEDs generated 45.2, 93.3, and 65.8 mW, respectively. For the 280 nm LED the lifespan of 50% was calculated to be 3000 h at a junction temperature of 30 °C. Their outcomes indicated that nitride-based DUV LEDs are more favorable as substitutes for mercury lamps (Figure 30). S. Sawyer observed that a possible degradation procedure might correspond to the diffusion of the Al atoms into the neighboring layers with a lesser Al mole fraction [85]. Consequently, the potential blockade for electrons shrinks, leading to the current rise and fall in electron concentration inside the quantum wells (QWs) in the light-emitting structure. Zhanhong Ma et al. studied the degradation of flip-chip 260 nm UVC-LEDs [81]. Their investigations indicated that: the voltage rise of the current in the reverse bias area and the low forward bias region, which was attributed to the rise in carrier tunneling stimulated by defects Figure 31.

**Figure 32:**
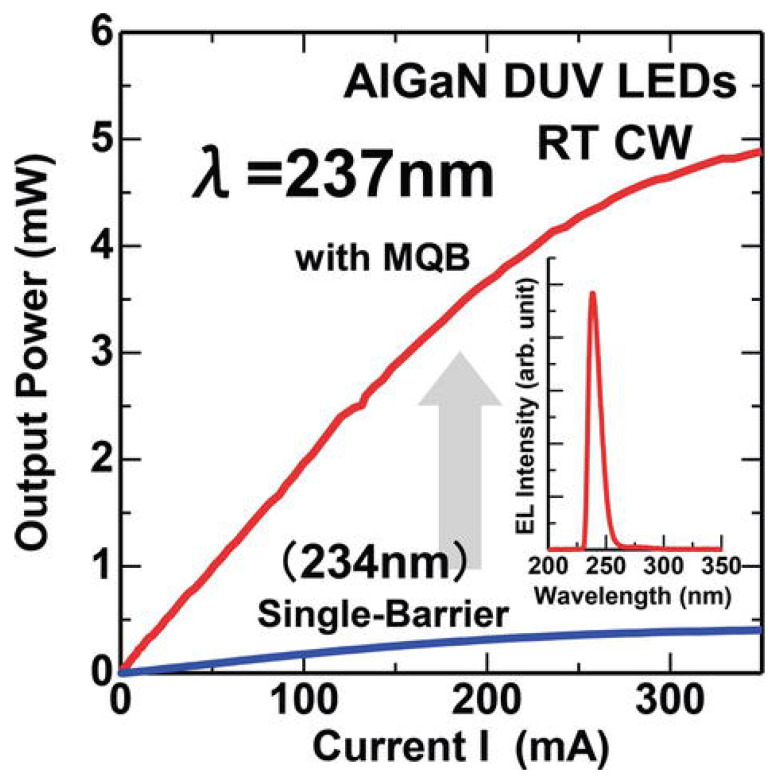
Increase in output power using MQB for 250-nm. Reproduced with permission. [87] Copyright 2015, Wiley.

Asif Khan et al. also worked on DUV LEDs with different geometries containing micro-pixel electrodes [86]. With this assembly, they got DUV LEDs emitting at 280 nm and lifetime more than 3000 h reducing the degradation and increasing the reliability. James R. Grandusky et al. fabricated pseudomorphic mid-UV-LED structures on AlN substrates, with low dislocation density, which led to a lower imperfection density throughout the n-Al_x_Ga_1-x_N and active area of the equipment. They made a comparison of pulsed operation and CW operation modes. Hideki Hirayama et al. provided a review of the evolution of growth of crystals based on AlN, the manufacturing of DUV-LEDs, and the corresponding competence [87]. They were capable of upgrading the yield power of DUV-LEDs remarkably by moderating the threading dislocations in AlN-crystals. Moreover, the utilization of doping, the insertion of MQB, and the usage of p-type electrodes having greater reflectivity were also included (Figure 32), So apart from the operational conditions setting there is a huge space where one can explore the constructional modifications which may lead to greater reliability and long lifetime devices.

## 8. AlGaN near UL-LEDs with Enhanced Reliability: A SARS-CoV-2 Solution

**Figure 33:**
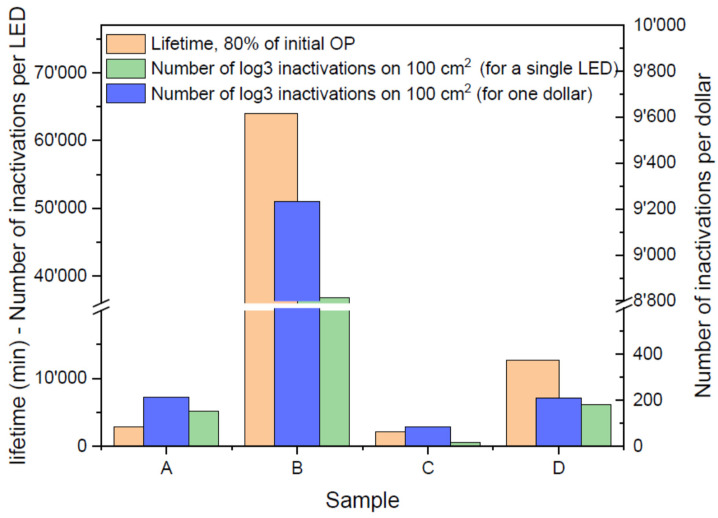
Summary of tested LED lifetime at 80% of initial optical power after stress at maximum current. Reproduced with permission. [89] Copyright 2022, MDPI.

The health issues triggered by SARS-CoV-2 have attracted researchers to improve the antiviral technologies, especially the use of ultraviolet light, being the most important technology [88] to unfailingly inactivate a wide range of microorganisms, including different viruses and bacteria. UVC irradiation has been found one of the most effective remedy against COVID-19 pandemic and urged the improvement of antiviral light-based technologies based on near UV LEDs. Compared to the indium gallium nitride based blue-emitting LEDs, the near UV LEDs need an enhanced bandgap, which is practically possible by means of the ternary compound of Al, Ga and nitride. However, all the development of UVC LEDs for antiviral systems strongly need an improved reliability of such devices. The previous efforts in this regard still produced a limited reliability of these devices and the degradation was possibly caused by an enhancement in Shockley–Read–Hall (SRH) recombination [89].That is why the use of UVC LEDs in continuous disinfection systems is still not suitable due to their limited reliability and lifetime. In this regard, N. Trivellin reported the reliability of the most recent commercial UVC LED devices. Their LEDs had been subjected to a stress test near the application limits and the reliability and characteristics have been analyzed. Upon finding a limited reliability, they suggested some relevant product design to improve the lifetime of near UV LEDs. They observed the dose required to observe an antiviral effect on SARS-CoV-2 virus and their results are shown in Figure 33 [89]. They concluded that the lifetime of near UV LEDs may still be sufficient for thousands of operations resulting from a single treatment of few minutes.

The ternary compound AlGaN provides the output wavelength as low as in the range of 230–240 nm; however, reaching this much shorter wavelength is difficult because of higher Al concentrations. The increased Al content results in the enhanced resistivity of the n-AlGaN layer which makes light extraction more difficult [29].

The researchers have worked out that once we move from 240 nm to 230 nm of wavelength, the emission intensity of an LED reduces approximately 10 times to that of original intensity [90] which is attributed to the c-plane light emission switching from TE to TM mode [91]. Furthermore, the higher Al content required for the n-Al*x*Ga(1-*x*)N layer causes an increased forward voltage (*V*f).

To improve the reliability of such near UV LEDs one effort is done by Johannes Glaab as mentioned in the earlier part of the review but one more effort was done by Akira et al. as they demonstrated long lifetime and high output power of UVC LEDs in the wavelength range from 230 nm to 237 nm. They demonstrated over 3600 h of lifetime operating at 20 mA for their LEDs which is the highest one reported in the literature for such a shorter wavelength of 240 nm. They attributed this enhanced lifetime to the reduction in the oxygen content in the high Al concentration layers that becomes the source of creation of point defects [29].

**Figure 34:**
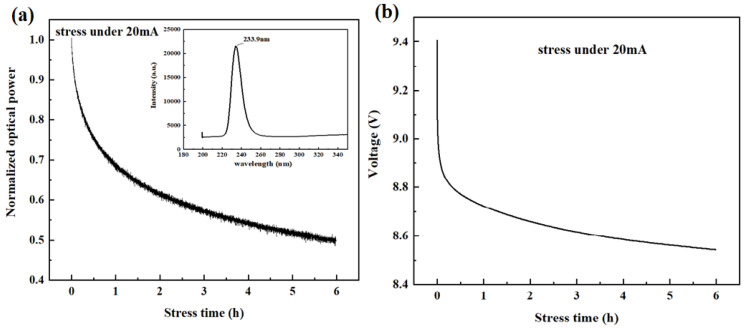
(**a**) Variation of optical power with aging time for 234 nm DUV LED at 20 mA current. Inset is the EL spectrum at 10 mA current, (**b**) The I-V curve under stress of 20 mA.

In the ongoing journey of reliability exploration, recently, we worked out the reliability of near UV LEDs for the wavelength of 234 nm. For the smaller wavelength DUV-LED, we have drawn the change of the optical power with aging time under 20 mA DC current of 303 K, as shown in Figure 34a. The inset of Figure 34a is the EL spectrum at 10 mA current, the luminescence peak of the device is at 234 nm. The optical power is normalized with the initial power before aging. Under a current stress of 20 mA, the optical power of the device rapidly dropped to 70% within 1 h, then after 6 h of aging time, the power dropped to 50% of the initial optical power.

Figure 34b shows the change in voltage with aging. With aging, the forward voltage of the device shows a downward trend, which from 9.4 V to about 8.6 V. The voltage of AlGaN DUV LEDs of other wavelengths reported in the previous literature were decreased after aging also, which may be related to the increased activation of the Mg dopant [67].

## 9. Degradation Mechanisms under Different Measurement Conditions

Among the different causes of degradation of an LED, the most contributing mechanism is the constant current stress which limit the lifetime of AlGaN-based UV-LEDs. This stress increases the rate of nonradiative recombination in the active region of the LED devices resulting in the decrease of their efficiencies. According to the previously reported measurement data and of this degradation can be ascribed to wo main reasons, namely, the spread of defects in active region of the LED devices and the diffusion of impurities toward the MQW region causing decreased internal efficiency [3].

**Figure 35:**
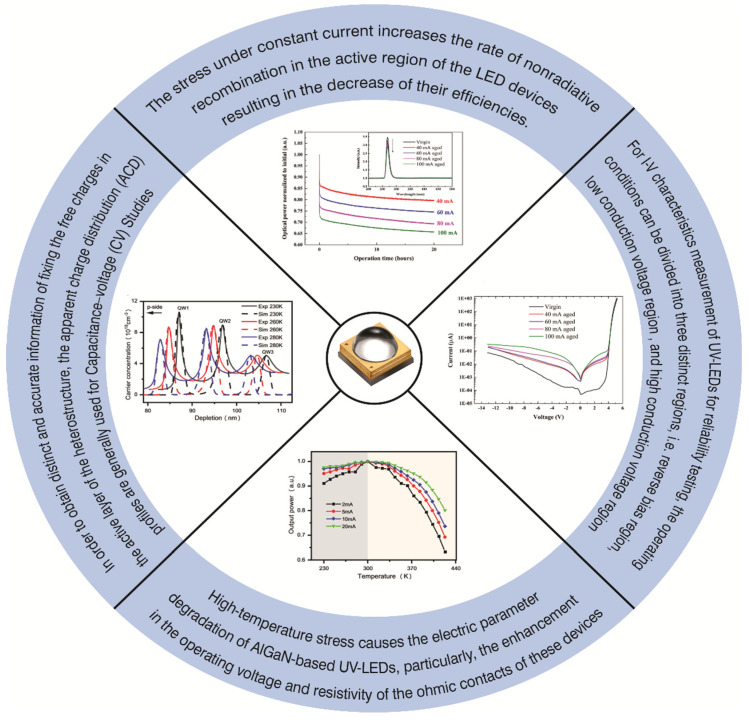
Reliability measurement scheme.

Based on our previous studies, there should be constant current stress measurements, for which one can choose different current values, such as 20 mA, 40 mA, 80 mA and 100 mA and observe the effect on the optical power before and after certain time intervals. The stress time could be from 24 h to 1000 h or more than that as reported in literature [40]. Relatively faster degradation in optical power results due to higher current densities, but follow the same trend as shown in the upper part of Figure 35. The inset of the figure also shows the measurements of PL spectra for unstressed sample and stressed sample for different current densities. As a general mechanism scheme, the degradation results in slight shift of the peaks, but the decrease in peak intensity reduces significantly with stressed current densities and aging time in PL studies. These measurements highlight the behavior that the defects and dislocation may be generated inside the active region without any change in Al composition [72].

For I-V characteristics’ measurement of UV-LEDs for reliability testing, the operating conditions can be divided into three distinct regions, i.e., reverse bias region, low conduction voltage region (~0–5 V), and high conduction voltage region (~5 V) as shown in Figure 35. Based on our previous work, we observed that the increase in current in high conduction voltage region did not change significantly compared to the other two regions, while in the low conduction voltage region, the current is usually correlated with trap-assisted tunneling [68]. In the reverse bias region, the leakage currents can be explained by thermally assisted multistep tunneling, that is, electron multistep tunneling from the p-side valence band (VB) to the n-side conduction band (CB) under thermal activation [77]. The leakage current at the reverse bias is usually smaller, which is related to the larger bandgap and more difficult tunneling [92]. In the reverse bias region, the leakage current is usually smaller and ascribed to the larger bandgap and this current can be explained by electron multistep tunneling from the p-side valence band to the n-side conduction band (CB) under thermal stress [77].

As mentioned earlier in Section 4, in order to obtain distinct and accurate information on fixing the free charges in the active layer of the heterostructure, the apparent charge distribution (ACD) profiles generally use capacitance–voltage (C-V) measurement. The carrier distribution versus depletion width is covered in the literature for different temperatures and a series of quantum wells and barriers are obtained. The bias conditions correspond to the depletion of quantum wells in the low forward voltage. An increase in current in the low forward bias region causes a decrease in carrier peak as the temperature increases. As the temperature decreases, the carrier concentration increases and both high and low temperatures have different temperature drooping mechanisms. If measurements are taken at low temperatures, there is a decrease in carrier peak and then at a certain threshold, it increases with the decrease in temperature. If the temperature is decreased, number of carriers bound in the well are increased, causing the carrier peak to rise slightly with this reduction in temperature.

High-temperature stress causes the electric parameter degradation of AlGaN-based UV-LEDs, particularly, the enhancement in the operating voltage and resistivity of the ohmic contacts of these devices. In one of our temperature-stress measurements, we described that the LEDs that had been fabricated with non-optimized contact technology led to the partial detachment of the contact layers as a result of high-stress conditions, ultimately increasing the device resistivity. The chromatic properties of white LEDs are strongly dependent on temperature, and stress at high-temperature levels induces a significant degradation. The temperature measurements could be optimized for two different scales—below 300 K and above 300 K (Figure 35). At low current densities, charge carriers mostly tunnel to the deep defect states in the space charge region causing nonradiative recombination. The thermal droop of UV-LED is thus caused by SRH recombination of thermally activated defects [56]. On the other hand, for high currents, thermal droop of UV-LED optical power is caused by the combination of SRH and active area electron leakage [93].

## 10. Concluding Remarks

We have presented here a review on near UV-LED performance and reliability. We have analyzed in detail the degradation effects due to different physical parameters, such as induced current, creation of defects, CV and IV behaviors and different device geometries of these LEDs subjected to stress conditions. The limited research done so far, according to our knowledge, has been reviewed for the findings and effects on degradation and is concluded as follows:

- There are several factors that affect the reliability and lifetime of an LED and the most prominent factor is the creation of point defects due to current stress applied for a given time and these point defects affect both the electronic and optical properties of the LED, especially at lower wavelength range, as mentioned above.
- The early aging of the LEDs is caused by the production of nonradiative centers in the active region of the devices as a consequence of lowering the internal quantum efficiency. When there is a consistent supply of operational current, the optical power is lowered. While the higher current densities accelerate the optical power degradation. The time dependence of optical power of near UV LEDs is found to be influenced by a scaling factor that is the cube of the operating current density.
- Below the startup voltage, the leakage current rises with the extension in the operational time. In comparison to higher currents, the low-current areas facilitate the changing of the emission power with respect to driving current.
- In the aging process, lowering of the emission intensity go along with the growth of the tunneling current, excessive nitrogen-vacancy concentration, and fragmentary compensation of the p-doping. However, the aging process remain unaffected on the lifespan of the carriers in QWs as well as p-cladding.
- The measurement of degradation of the electrical characteristics of the LEDs can be ensured by high-temperature stress. Also, it helps in the rise of the operating voltage of the devices. The degradation rate was found to rise up with increasing junction temperature level. The widening of the active region (i.e., p-contact) can raise the reliability level. The modified carrier distribution i.e., excessive QWs in the active region, can be an effective strategy for the extension of the lifetime.
- The production of defects generates the energy levels within the bandgap. This allows the electrons from these levels to jump up to the conduction band (CB), giving rise to a photocurrent, which is the main characteristic of presence of imperfections in optoelectronic devices.
- For devices with enhanced Al content, emission bands associated with the optical transitions in the cladding give rise to nitrogen vacancies. The evolution of the emission band indicates the stronger influence of the N vacancies in aging process, as it facilitates the production of defects and high-conductivity channels.
- The current lower than the corresponding startup voltage rises with the square root of time dependence. This suggests the occurrence of a diffusion process, using point defects as a source for giving rise to nonradiative recombination in the LED.
- The substrates containing the heteroepitaxial growth of near UV-LEDs have large lattice imperfections causing dislocation densities and material defect concentration can be larger, so careful optimization of the growth and treatment processes are crucial for obtaining high device performance.
- The degradation shows its manifestation in *C*–*V* measurements where the stress causes modification in the charge distribution of the active layer, which creates defectiveness. Stress introduces a charge redistribution in the active layer of the devices, and specifically, a consistent increase in charge concentration in the quantum-well region. This suggests the production and transmission of charged centers within the quantum well region, and explains the influence of degradation process on the position of the semiconductor region.
- In the forward-bias range, the rising aging current reduces the capacitance. The lowering of the peak capacitance with rising stress currents suggests the broadening of the depletion widths for unstressed devices. The barrier capacitance governs the whole capacitance: the LED works under the influence of reverse-bias voltage, whereas for forward-bias voltage, the larger portion is contributed by diffusion capacitance. As the reverse-bias voltage falls, the micro-LED capacitance rises, which points out the surface of the space charge region movement.
- The nonlinearity in the I-V characteristic was not only caused by the p-n junction but also from the non-ohmic nature of the p- and n-contacts. This displays approximately the reverse-biased Schottky behavior. DC stress can also give rise to reverse bias as well as the low-forward bias current components. During stress, the current rise for a given voltage is less than the startup value. Eventually, the I-V characteristic curves indicate the lowering of the drive voltage throughout stress treatment at the peak voltages. Throughout the aging interval, the reverse leakage current rises for the corresponding reverse bias region. However, the leakage current achieves a stable value with a logarithmic dependence on stress duration.

## Figures and Tables

**Table 1:** A comparison of AlGaN-based UV-LED manufacturing techniques.

Group	Technique	Substrate	EQE	Wavelength	Output Power	Year	Ref
Ajmal Khan et al.	graded stacks of AlGaN buffer layer	Sapphire	4.4%	310 nm 295 nm	7.1 mW 13 mW	2019	[25]
Shucheng Ge et al.	nanoimprint lithography and top-down dry etching	Sapphire	2.03%	277 nm	-	2019	[26]
Ajmal khan et al.	highly reflective Ni/Al p-electrode and p-MQB EBL	Sapphire	4.7%,	310 nm	29 mW	2020	[16]
Luca Sulmoni et al.	V/Al-based n-contacts with different Al mole fractions	AlN bulk substrate	0.12%	232 nm	120 μW	2020	[27]
Tien-Yu Wang et al.	AlGaN and GaN sidewalls were turned into Al_x_Ga_2–x_O_3_ and Ga_2_O_3_ sidewalls, respectively, by thermal oxidation	Sapphire	0.76%	280 nm	0.69 mW	2022	[28]
Akira Yoshikawa et al.	improved n-metal electrodes	AlN single crystal substrates	-	237 nm, 235 nm, 233 nm and 230 nm	2.2 mW, 1.9 mW, 1.5 mW and 1.2 mW	2019	[29]
Yukio Kashima et al.	introducing a highly reflective photonic crystal into the surface of the p-AlGaN contact layer	Sapphire	10%	283 nm	>20 mW	2018	[20]
Dong Liu et al.	employing p-type Si	single crystal bulk aluminum nitride (AlN)	0.03%.	229 nm	160 ηW	2018	[17]
Liang Lu et al.	developing composite last quantum barrier (CLQB)	Sapphire	-	310 nm	Increased by 30%	2021	[30]
Ray-Hua Hrong et al.	Zinc gallate thin films as the p-type transparent contact layer	Sapphire	5.5%	280 nm	~12 mW	2017	[31]
Chunshuang Chu et al.	polarization induced positive sheet charges at the last quantum barrier (LQB)/p-EBL interface.	-	3.5%	280 nm	~20 mW	2019	[32]
Youn Joon Sung et. al	using a highly reflective ITO/Al *p*-type electrode.	-	6.68%	277.6 nm	630 mW	2019	[33]
Su-Hui Lin et al.	Introducing a reflective passivation layer (RPL) consisting of HfO2/SiO2 stacks	Sapphire	3.09%	280 nm	125.24 mW	2021	[18]
Michiko Kaneda et al.	fabricated on n-AlGaN templates	AlN templates on sapphire	3.5, 3.9, 6.1, and 6.0%	266, 271, 283, and 298 nm,	~26 mW	2017	[34]
Takayoshi Takano et al.	a transparent AlGaN:Mg contact layer, a Rh mirror electrode, an AlN template on a patterned sapphire substrate, and encapsulation resin.	Sapphire	20%	275 nm	44.2 mW	2017	[35]
Zi-Hui Zhang et al.	specifically designed superlattice p-type electron blocking layer (p-EBL).	Sapphire	~3.4%	270 nm	~24 mW	2018	[21]

**Table 2:** Summary of the operating conditions of current stress and related outcomes.

Research Group	Temperature	Required Time	Current	Voltage	Intensity/Voltage/Current	Reason(Degradation Cause)	Ref
A. Pinos et al.	600–1000 K	86 h	100 mA	(~3.5 V)	increase tunneling current, decrease emission intensity	formation of tunneling conductivity channels	[50]
Matteo Meneghini et al.	180–250 °C	(100–1000 h)	20 mA	−5 V to 3 V	current decreasesvoltage increases	increase of nonradiative recombination paths	[39]
Matteo Meneghini, et al.	>80–100 °C	2000 h	1 A	2 to −2 V	current increasesvoltage decreases	Degradation rate increase with increasing junction temperature level.	[3]
Matteo Meneghini et al.	35–85 °C	1200 h	20 mA	4 V to 6 V	current decreases	generation/propagation of defects in the active region of the devices	[40]
Craig G. Moe et al.	57 °C and 184 °C	64 h	20 and 75 mA		current decreases	Increased nitrogen vacancy formation	[46]
Johannes Glaab et al.	20 °C	1000 h	100 mA	19.9 V	current decreasesvoltage increases	Nonradiative recombination centers occurrence in or around the active region.	[36]
Huixin Xiu et al.	135 °C	50 h	20 mA	6.367 V	intensity decreasestunneling current increases	decrease of the leakage current	[47]
N. Trivellin et al.	15 °C to 85 °C.	2000 min	350 and 500 mA	6.5 V	current increasesvoltage decreases	new emission peak arose	[48]
Lilin Liu, et al.	25 °C	1000 h	600 mA	−3 V to 3 V	tunneling current increases	damaging the active layers and improving the p-type conductivity	[57]
Jan Ruschel et al.	25 °C	1000 h	50 to 300 mA	9 V	current increases	Degradation rate independent of the current density.	[51]

**Table 3:** Summary of the C-V response to the stress.

Literature Survey	Frequency Range	Required Time	Voltage Range	Capacitance/ Voltage/ Current	Reason (Degradation Cause)	Ref
Matteo Meneghini et al.		270–500 h	5.4 V	voltage increases	Increase in the defectiveness of the active layer of the LEDs.	[25]
Matteo Meneghini et al.		20–1000 h	2.5 V	current decreases	Increase in defect-related radiative recombination components.	[32]
Craig G. Moe et al.	1 MHz	0–64 h		current decreases	wider depletion region which extends further into the *p*-layer after electrical degradation	[17]
Our Group results	200 Hz–2 MHz	0.5 h		capacitance increases	Increase in the trap centers	[43]
Matteo Meneghini et al.		250 h	(−8–0) V	current decreases	Increase of the nonradiative recombination rate.	[51]
Our Group results	1 MHz		(0–5) V	capacitance increases	Increase of nonradiative paths.	[42]
Pradip Dalapati et al.		100 h	1.46 V	capacitance increases	narrowing of depletion width	[45]
Zhanhong Ma et al.	1 MHz	400 min	−20 V to 5 V	capacitance decreases	decrease of the effective carrier concentration, because of the increased defect during the operation	[48]

**Table 4:** A summary of the I-V response to the stress.

Literature Survey	Required Time	Current Range	Voltage Range	Voltage/ Current	Reason (Degradation Cause)	Ref
Johannes Glaab et al.	1000 h	100 mA	19.9 V to 20.6 V	voltage increases	nonlinearity in the I-V characteristic	[36]
Lilin Liu et al.	124.5 h 274 h 487 h	20 mA	−6 V to 6 V	current increases	Shows ohmic behavior.	[57]
Matteo Meneghini et al.	2–1064 h	20 mA	−8 V to 4 V	current increases	good stability of ohmic contact and resistivity of neutral regions over stress time	[40]
Our Group results	24 h	80 mA	1–2 V	current increases	activation of open-core dislocation in the active region	[73]
Pradip Dalapati et al.	100 h	60 mA	−5 to −10 V	current decreases	improvement in the net charge concentration	[75]
Our Group		40–100 mA	−12 V to 6 V	current increases	defect-assisted tunneling process	[72]
Zhanhong Ma et al.	400 min		−10 V to 2 V	voltage decreases	defects increase within the active region	[78]
Desiree Monti et al.	1–1000 h	≤2 nA	0 to ∼4 V	current increases	generation of mid-gap states	[58]
F. Piva et al.	(t < 1000 min) (t > 1000 min)	250 mA 350 mA	(V < 4.5 V), 1 V < V < 3 V	current increases	increase in the density of defects in the active region	[67]
Desiree Monti et al.	20–50 h	250 mA	0 V to ~4 V	current increases	increase in the carrier generation components	[66]
Zhanhong Ma et al.	0–150 h	0.05 A	0 to 4.5 V	current increases	forming parasitic current paths across the active region	[81]

## Data Availability

Not applicable.
